# Optimizing indices of atrial fibrillation susceptibility and burden to evaluate atrial fibrillation severity, risk and outcomes

**DOI:** 10.1093/cvr/cvab147

**Published:** 2021-04-29

**Authors:** Giuseppe Boriani, Marco Vitolo, Igor Diemberger, Marco Proietti, Anna Chiara Valenti, Vincenzo Livio Malavasi, Gregory Y H Lip

**Affiliations:** 1 Cardiology Division, Department of Biomedical, Metabolic and Neural Sciences, University of Modena and Reggio Emilia, Policlinico di Modena, Via del Pozzo, 71, 41124 Modena, Italy; 2 Liverpool Centre for Cardiovascular Science, University of Liverpool and Liverpool Heart & Chest Hospital, Liverpool, UK; 3 Clinical and Experimental Medicine PhD Program, University of Modena and Reggio Emilia, Modena, Italy; 4 Department of Experimental, Diagnostic and Specialty Medicine, Institute of Cardiology, University of Bologna, Policlinico S. Orsola-Malpighi, Bologna, Italy; 5 Department of Clinical Sciences and Community Health, University of Milan, Milan, Italy; 6 Geriatric Unit, IRCCS Istituti Clinici Scientifici Maugeri, Milan, Italy; 7 Aalborg Thrombosis Research Unit, Department of Clinical Medicine, Aalborg University, Aalborg, Denmark

**Keywords:** Atrial fibrillation, AF susceptibility, Stroke, AF burden

## Abstract

Atrial fibrillation (AF) has heterogeneous patterns of presentation concerning symptoms, duration of episodes, AF burden, and the tendency to progress towards the terminal step of permanent AF. AF is associated with a risk of stroke/thromboembolism traditionally considered dependent on patient-level risk factors rather than AF type, AF burden, or other characterizations. However, the time spent in AF appears related to an incremental risk of stroke, as suggested by the higher risk of stroke in patients with clinical AF vs. subclinical episodes and in patients with non-paroxysmal AF vs. paroxysmal AF. In patients with device-detected atrial tachyarrhythmias, AF burden is a dynamic process with potential transitions from a lower to a higher maximum daily arrhythmia burden, thus justifying monitoring its temporal evolution. In clinical terms, the appearance of the first episode of AF, the characterization of the arrhythmia in a specific AF type, the progression of AF, and the response to rhythm control therapies, as well as the clinical outcomes, are all conditioned by underlying heart disease, risk factors, and comorbidities. Improved understanding is needed on how to monitor and modulate the effect of factors that condition AF susceptibility and modulate AF-associated outcomes. The increasing use of wearables and apps in practice and clinical research may be useful to predict and quantify AF burden and assess AF susceptibility at the individual patient level. This may help us reveal why AF stops and starts again, or why AF episodes, or burden, cluster. Additionally, whether the distribution of burden is associated with variations in the propensity to thrombosis or other clinical adverse events. Combining the improved methods for data analysis, clinical and translational science could be the basis for the early identification of the subset of patients at risk of progressing to a longer duration/higher burden of AF and the associated adverse outcomes.

## Graphical Abstract

**Figure:**
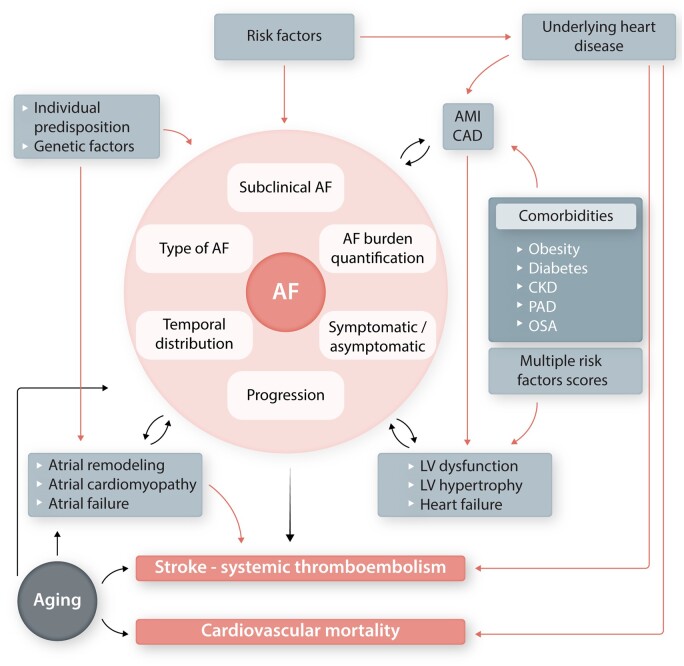

**This article is part of the Spotlight Issue on Atrial Fibrillation.**

## 1. Introduction

Atrial fibrillation (AF) is an arrhythmia with a heterogeneous pattern of presentation, in terms of symptoms, duration of episodes, time spent in AF, evolution over time, and dependence on underlying heart disease, risk factors, and comorbidities. Clinical management of AF could markedly benefit from translational research and should provide important feedback on how to direct basic research to a ‘bench to bedside and back again’ process. In this review, we will summarize some updated data on AF susceptibility and characterization, considering AF burden and its dynamic changes, including relation to clinical factors and indices that may condition AF onset and burden, as well as patients’ outcome(s). We specifically focused on a series of clinical factors and comorbidities that are related to AF susceptibility in terms of AF incidence, variable AF burden, and tendency for progression to permanent AF.

## 2. Arrhythmia burden: dynamic relationships, arrhythmia progression, and clinical implications for stroke risk

AF is associated with a substantial risk of mortality and morbidity from stroke and thromboembolism (TE)^1–4^ and risk stratification for predicting stroke and TE is a key requirement of clinical decision-making.^5^ The CHA_2_DS_2_-VASc score is a simple stroke risk stratification tool targeted to identify patients at low risk of stroke/TE,^6^ thus, providing a guide for stroke prevention with oral anticoagulants (OACs).^1^^,^^5^^,^^7^ Different biomarkers have been integrated into several risk scores for stroke and bleeding prediction in the AF population (e.g. ABC-stroke, ATRIA and ABC-bleeding), adding a modest, although statistically significant, improvement to conventional clinical-based scores.^8^ The addition of biomarkers may represent an opportunity to fine-tune indices of AF susceptibility. However, biomarkers alone may fail to predict specific outcomes *per se* and need to be integrated with clinical elements to provide more targeted care. Many researchers have tried to investigate which parameters could allow an improvement in the prediction of stroke/TE on top of the CHA_2_DS_2_-VASc score, by including assessments related to the specific type of AF as an indirect estimate of the time spent in AF, or by measuring the burden of AF as a consequence of rhythm monitoring.^1^^,^^5^ Although the term ‘AF burden’ has been used in the past with different meanings,^9^^,^^10^ there is now an agreement in defining it as the overall time spent in AF during a specified period. This term has also been adopted to describe the temporal dynamic pattern of AF in regard to the presence and duration of AF episodes, especially when data from the continuous monitoring of an implanted device are available.^11^^,^^12^ Based on the clinical presentation of AF, and taking into account clinical data on arrhythmia duration, different clinical subtypes of AF have been proposed, independently of symptoms: first diagnosed AF, paroxysmal AF, persistent AF, long-standing persistent AF, and permanent AF.^5^ This classification is based on the clinical presentation, but only partially reflects AF duration given the high proportion of possible asymptomatic AF episodes, thus requiring detection and diagnosis based on electrocardiogram (ECG) recordings of variable duration, corresponding to a variable intensity of monitoring.^13^ From this perspective, the 4S-AF (Stroke risk, Symptom severity, Severity of AF burden, Substrate severity) scheme recently proposed by the 2020 European Society of Cardiology (ESC) guidelines on AF, has the potential to improve the characterization of AF patients, moving towards a more comprehensive and structured AF characterization rather than a single-domain AF classification.^5^^,^^14^ Traditionally, stroke risk stratification in AF is related to patient-level risk factors rather than AF type, and the widely used scores for risk stratification do not include the characterization of AF type. Current guidelines recommend that decision-making on anticoagulation should be independent of specific AF type.^1^^,^^5^ However, there is increasing interest in assessing if the quantification of AF may allow for a more precise assessment of stroke risk, beyond the traditional risk stratification based on a binary approach, i.e. AF present vs. AF absent.^15^

The simplest approach to this topic was based on evaluating the risk of stroke in paroxysmal vs. non-paroxysmal AF. Observational studies exploring real-world practice showed that non-paroxysmal AF types are common and predominant in daily practice.^16^^,^^17^ For example, in the EURObservational Research Programme on AF (EORP-AF) General Pilot Registry,^16^ patients with paroxysmal AF accounted for 27% of the cohort and were younger, with a lower prevalence of heart disease and major comorbidities, compared with non-paroxysmal AF patients. In the registry, patients with non-paroxysmal AF had a worse outcome at 1 year, in terms of all-cause mortality due to a more severe clinical profile. On the other hand, the risk of stroke at 1 year was relatively low, with no differences between paroxysmal and non-paroxysmal AF, perhaps reflecting the high rates of anticoagulation applied in this cohort.^16^ Vanassche *et al*.^18^ evaluated the rates of stroke and systemic embolism in 6563 aspirin-treated AF patients from the ACTIVE-A and AVERROES databases and found that permanent AF was the second independent strongest predictor of stroke, after prior stroke or transient ischaemic attack (TIA).

A systematic review and meta-analysis of 12 studies on nearly 100 000 patients evaluating the risk of stroke/TE in paroxysmal vs. non-paroxysmal AF patients, indicated that the risk of stroke is significantly higher in non-paroxysmal AF when compared with paroxysmal AF. However, the heterogeneity in study design, treatments and ascertainment of outcomes imposes caution in the interpretation of results.^17^

Since patients with non-paroxysmal AF spend more time in AF compared to those with other AF types, it can be hypothesized that the overall temporal burden of AF is related to an increased risk of stroke. However, it is uncertain whether the AF pattern is truly an independent predictor of stroke beyond the effect of the many potential unmeasured confounders, rather than a reflection of a different patient profile, in terms of risk factors and comorbidities. Moreover, AF has a dynamic nature and variable evolutions, including the possibility to be progressive, with reported rates of progression to permanent AF in up to 25% of paroxysmal AF patients, depending on patient age, presentation at baseline, underlying heart disease, and treatments/interventions.^9^ Among patients with stroke risk factors recruited from the general population, progression to permanent AF was observed in around one patient out of six (i.e. in 16%) continuously monitored by an implantable loop recorder (ILR) for a median of 40 months in the LOOP study.^19^ In an individual patient perspective, there is a need for improving the prediction of the evolution of AF along with duration, since both progression from short to long duration AF (including permanent AF) and reduction up to remission of AF can be observed. The heterogeneity of AF behaviour and the variable temporal dynamics of its potential progression or regression should be better investigated, adding different variables, preferably related with the atrial substrate beyond the quite expected clinical predictors of AF evolution [hypertension, heart failure (HF), previous stroke].

**Figure 1:**
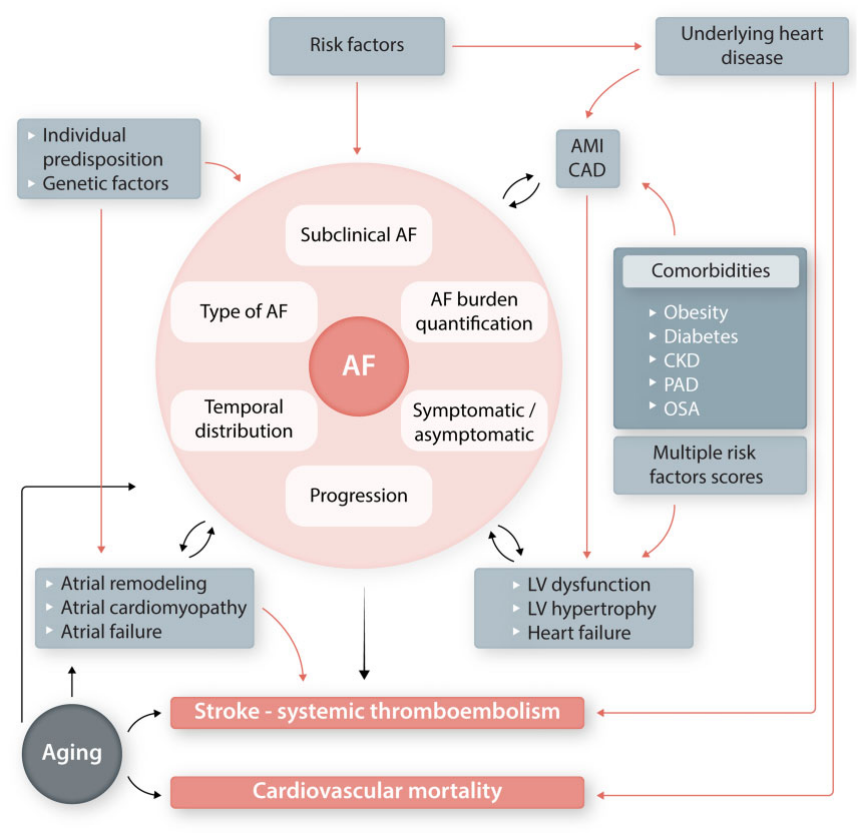
The complex interplay between atrial fibrillation, underlying atrial and ventricular factors, comorbidities, and adverse outcomes (stroke, cardiovascular mortality). AF, atrial fibrillation; CAD, coronary artery disease; CKD, chronic kidney disease; LV, left ventricular; OSA, obstructive sleep apnoea; PAD, peripheral artery disease.

Given the limitations to quantify AF on the basis of clinical parameters and intermittent monitoring, additional insights come from studies on patients implanted with cardiac implantable electrical devices (CIEDs). CIEDs with an atrial lead allow a continuous monitoring of the atrial rhythm, coupled with the storage of data on atrial tachyarrhythmias and AF episodes presence, duration, time of occurrence, and temporal distribution, thus resulting in a precise quantification of the burden of atrial arrhythmias. For episodes of atrial tachyarrhythmias detected by CIED, referred to as atrial high-rate episodes (AHREs) or subclinical AF, the diagnostic accuracy is highly reliable when episodes ≥5 min in duration are considered.^13^^,^^20^^,^^21^ According to the ESC Guidelines on AF,^5^ subclinical AF is defined as AHRE confirmed to be AF or atrial tachyarrhythmia, or AF documented by an insertable cardiac monitor or a wearable monitor and confirmed to be AF by tracing analysis. It has been shown that AHRE or subclinical AF episodes lasting at least 5–6 min are associated with both an increased risk of subsequent onset of clinical AF [odds ratio (OR) 5.7, 95% confidence interval (CI) 4.0–8.0] and an increased risk of stroke/TE (OR 2.4, 95% CI 1.8–3.3).^22^ Preliminary analysis also suggests that the dose–response relationship between AF burden and the risk of stroke/TE may be non-linear.^23^ All of the findings related to AHRE have to be interpreted, taking into account that they derived from selected populations, characterized by the need for implanting a device for bradycardia or sudden death prevention or those with HF and an indication for cardiac resynchronization therapy. Since the risk of stroke/TE for subclinical AF/AHRE may be around half of the risk associated with clinical AF,^22^ the threshold of AF burden above 5–6 min at which the risk of stroke is markedly increased (and favouring the risk-benefit ratio for OACs) is still undefined.^24^^,^^25^ This question is under evaluation in clinical trials.^26^^,^^27^ However, a burden of at least 24 h seems to be associated with a significantly higher risk of stroke, compared to patients without AHRE.^28^ AF burden, as detected by CIEDs, is a dynamic process with frequent transitions from a lower to a higher maximum daily burden, thus making it appropriate to monitor the temporal evolution of daily burden in association with the clinical profile.^29^ In patients presenting with clinical paroxysmal AF, the temporal pattern of AF as assessed by continuous monitoring through CIEDs is heterogeneous, with AF recurrences and progression to episodes of longer AF duration related to associated comorbidities and a higher CHA_2_DS_2_-VASc score.^30^ Depending on the type of study and length of follow-up, atrial premature complexes and repetitive atrial complexes (also referred to as ‘micro-AF’), may be associated with an increased risk of stroke and may predict AF detected by prolonged monitoring.^31^ More detailed analysis of AF burden dynamics and temporal distribution of AF episodes can be performed, such as analysis of AF density^32^ that could provide a more granular characterization of AF, with potentially novel implications for assessing the relationship with stroke risk and atrial remodelling. In studies on CIEDs, both the burden of AF and the duration of AF episodes have been assessed and the two variables appear correlated to each other, although a direct association with stroke/TIA is not validated^33^ unless variable combinations with CHADS_2_ or CHA_2_DS_2_-VASc are taken into consideration.^21^ Notably, these risk stratification scores were first proposed in the setting of clinical AF, and the reported incidence rates of stroke events were lower for AHRE compared with clinical AF, although with an increased risk of stroke associated with increasing stroke risk factors.^34^^,^^35^ Recently, the LOOP study analysed the burden of AF in elderly patients with cardiovascular risk factors, by assessing the role of enhanced AF detection through an ILR.^19^^,^^36^ The study found that ILR may detect AF ≥6 min among the elderly with an incidence rate between 22% at 18 months^37^ and 40% at 30 months.^38^ Moreover, during the follow-up around 16% of the patients presented AF episodes lasting ≥24 h that were preceded in 85% of the cases by shorter AF episodes, indicating an individual tendency to AF progression, with some variability in time relationships.^19^ Hence, there is a need for more powerful predictors of AF burden and identification of the subset of patients at risk of progression to longer duration/higher burden, also taking into account the overall clinical picture and risk factors (hypertension, HF, previous stroke). Up until now, the detection and monitoring of AF has been traditionally based on medical tools and devices, but recently the field has been revolutionized by advanced digital technologies, applied both in practice and in trials, with the emerging use of wearables and healthcare apps usually proposed as direct to consumer products.^39^ At present there is still a need to coordinate and organize the use of wearables in daily practice.^40^ There are many future opportunities, including virtual clinical trials, integration with electronic medical records, and data elaboration with artificial intelligence to expand the possibilities of detecting and monitoring AF beyond the traditional settings. The temporal relationship between AF burden and stroke could imply periods of increased risk, as suggested by the case-crossover analysis of Turakhia *et al*.^41^ However, the temporal dissociation between the presence of AF and the occurrence of stroke, found in many patients with a CIED suggests that AF may act either as a true risk factor for stroke (with a causal relationship linked to cardioembolism) or as a simple marker of risk, with stroke not strictly related to AF-related cardioembolism^10^^,^^42^^,^^43^ (*Figure 1*).

## 3. AF and the cardiac substrate: clinical implications and translational perspectives

There is a need for improving the characterization of AF in individual patients, as recently suggested with the 4S-AF Scheme.^5^^,^^14^ This requires knowledge of the role that cardiac factors and comorbidities may exert in increasing AF susceptibility, in conditioning AF incidence, AF burden and AF progression, as well as in modulating AF-associated outcomes in a complex interplay of mutual interactions (*Figures 1 and* *2*). Indices of AF susceptibility based on the AF burden pattern and dynamic changes that could be considered for improved patient characterization in the 4S-AF scheme are shown in *Table 1*.

**Figure 2:**
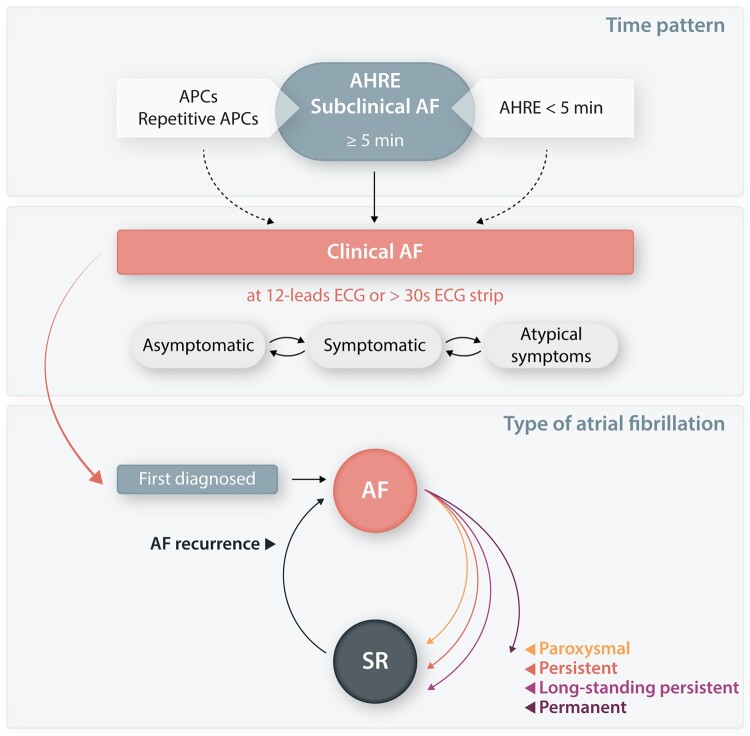
Time pattern and type of atrial fibrillation. AF, atrial fibrillation; AHRE, atrial high-rate episodes; APC, atrial premature contraction; min, minutes; SR, sinus rhythm.

**Table 1:** Indices of AF susceptibility based on AF burden pattern and dynamic changes that could be considered for improved patients’ characterization in the 4S-AF scheme

• New-onset AF burden
• Daily AF burden
• Monthly AF burden
• Increase in AF burden
• Decrease of AF burden up to zero burden
• AF burden aggregation (AF density)
• Subclinical AF clustering
• Evolution of subclinical AF to clinical AF (paroxysmal/persistent/permanent)
• Clinical AF distribution in a specific period of time
• Clinical AF episodes clustering

AF, atrial fibrillation.

### 3.1 Atrial cardiomyopathy and atrial function

#### 3.1.1 Left atrial cardiomyopathy as a factor facilitating AF onset

Atrial cardiomyopathy and atrial structural remodelling constitute the background for incident AF and for its progression to more sustained forms. Given the variety of underlying structural changes and the dynamic nature of structural atrial remodelling, a specific definition of atrial cardiomyopathy is challenging. In 2016, a consensus document from the European Heart Rhythm Association (EHRA)/Heart Rhythm Society (HRS)/Asia Pacific Heart Rhythm Society (APHRS)/SOLAECE proposed a pathophysiological classification focused on histological changes, recognizing four classes according to the leading mechanism of structural remodelling (cardiomyocyte changes, fibrosis, non-collagen infiltration, or a combination of those).^44^ Notably, the term ‘atrial failure’ has been recently proposed as an independent, clinically relevant entity beyond AF and HF, defined as ‘any atrial dysfunction causing impaired heart performance and symptoms and worsening quality of life or life expectancy, in the absence of significant valvular or ventricular abnormalities and distinct from either atrial remodelling and atrial cardiomyopathy’.^45^

Since an atrial biopsy is not routinely available, the most reliable indexes of atrial remodelling in clinical practice are atrial size and function. The role of left atrial (LA) diameter (LAD) in predicting incident AF was recognized in both the Framingham population, where the risk of incident AF raised by 30% for each 5 mm increase in LAD [adjusted hazard ratio (HR) 1.31; 95% CI 1.14–1.52],^46^ and in the Cardiovascular Health Study, in which LAD was an independent predictor of new-onset AF (relative risk 1.74, 95% CI 1.44–2.11).^47^ However, LA volume provides a more accurate assessment of LA size and better predicts AF development, since a 30% increase in LA volume was found to carry an independent 43% additional risk of AF onset, compared to a 38% additional risk related to increasing LAD.^48^ Moreover, the joint implications of both LAD and LA volume on the cumulative risk of AF were incremental to each other and to clinical factors.^48^ Even in sinus rhythm patients, LA volume, indexed for the body surface area (LAVI), was greater in predicting a composite of first-diagnosed AF, stroke or mortality than LA area or diameter (area under the curve for LAVI 0.71 vs. 0.64 for LA area vs. 0.59 for LAD).^49^

The predictive power of atrial remodelling is even greater when considering LA function and specifically, LA emptying fraction (LAEF) as firstly reported in a prospective study on sinus rhythm patients.^50^ The study found that LAEF ≤49%, measured by echocardiography, carried a more than a five-fold increased risk for new AF/atrial flutter than LA volume >38 mL/m^2^, independently of clinical risk factors.^50^

In recent years, great developments on clinical imagery, such as LA strain-derived quantification of LA function, could fill the gap between biological knowledge and clinical care of the arrhythmia. The role of LA strain in AF is promising since there is increasing evidence that LA strain could be used as a valid predictor of AF occurrence and recurrence,^51^ and potentially as a predictor of thromboembolic events. Additionally, LA strain may finely characterize the tissue composition including fibrosis, fat accumulation, and pave the way for the search of new biomarkers in high-risk patients. For example, atrial function assessment by speckle-tracking strain echocardiography was a strong predictor of incident AF in the Cardiovascular Health Study. As a marker of pre-clinical atrial dysfunction, LA strain strongly predicted new-onset AF, independently of traditional LA measurements and clinical risk factors.^51^

#### 3.1.2 Atrial cardiomyopathy, atrial function and burden of AF

Higher AF burden and LA remodelling are known to be closely intertwined.^52^ LAD as a continuous variable was a major determinant of AF progression in the YOUNG-AF study.^53^ Moreover, in a contemporary cohort of AF patients, LA enlargement was independently associated to AF progression and the addition of at least moderate LAVI to HATCH score significantly improved the prediction of evolution to permanent AF.^54^

#### 3.1.3 Atrial function as a factor modulating outcomes

Atrial enlargement was originally considered as a significant predictor of stroke and recurrences,^55–57^ but further evidence from the Cardiovascular Health Study questioned this relation.^58^ In a large retrospective study on 8679 unselected patients, LA enlargement was found in almost half of the population, and an increasing stroke prevalence was observed with enlarging LA (16% normal, 19% mild, 20% moderate, and 22% severe). The association between LA enlargement and stroke failed to reach statistical significance (adjusted OR 0.98; 95% CI 0.86–1.12), while AF was confirmed as the true thromboembolic risk factor (adjusted OR 1.34, CI 1.15–1.56).^59^

#### 3.1.4 Translational implications

Atrial cardiomyopathy encompasses a broad variety of underlying conditions and histological abnormalities, often poorly understood. Structural atrial remodelling certainly plays a central role in promoting AF onset and maintenance and its recognition, mainly in the early subclinical phase, may promote additional prevention strategies and individualized management. However, the relationship between AF and atrial cardiomyopathy appears to be bidirectional and further studies are needed to understand the dynamics and the determinants of LA structural changes, also with regard to the implications for therapeutical management. The relationship between the cumulative AF burden and atrial function is not well established and requires further investigation. Both animal and human studies have found that cardioversion of AF episodes of brief duration (<1 h) either is not associated with a depression of atrial contractile function (i.e. ‘atrial stunning’) or results in an atrial stunning that resolves within a few minutes^60^^,^^61^ Conversely, atrial stunning is well documented after conversion of AF lasting 1–2 weeks, with the resumption of contractile function in 1–2 days, while in the case of persistent AF post-cardioversion atrial stunning may resolve within 1–4 weeks in most patients.^62^ It is unknown what is the effect of repeated episodes of AF or AHRE lasting several hours on atrial contractility and if the cumulative time spent in AF may impair the recovery of atrial function after sinus rhythm resumption.^60^ Atrial fibrosis is an integral component of atrial remodelling in AF and is associated with progressive atrial dilation, reduced mechanical function and AF susceptibility. However, the burden of atrial fibrosis does not always directly correlate to the extent of AF burden, as clinically defined on the basis of paroxysmal or persistent AF^63^ thus raising the question of what amount of atrial burden may trigger the complex network of pathophysiological mechanisms leading to fibrosis^64^ and which is the extent of individual variability in development of fibrosis.

## 4. AF and comorbidities: clinical implications and translational perspectives

**Figure 3:**
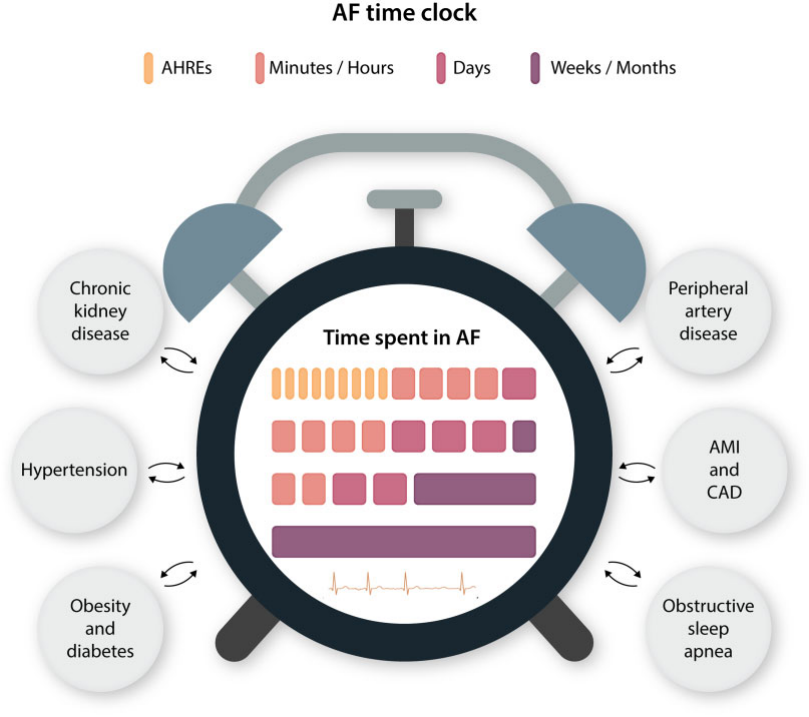
Atrial fibrillation time clock: the effect of a series of comorbidities affecting AF temporal pattern. AF, atrial fibrillation; AMI, acute myocardial ischaemia; CAD, coronary artery disease.

Many comorbidities are affecting the development and evolution of AF, as well as the clinical course of AF patients, with influences on AF temporal patterns and AF burden. Different comorbidities are recognized but they are not exactly weighted in quantitative terms, also in consideration of the mutual interactions that exists. The effect of the most common comorbidities affecting AF incidence, temporal pattern and outcomes are shown in *Figure 3*, *Table 2*^S1–S18^ and *Table 3*^S19–S42^ (Tables’ references are listed in the Supplementary material online). Potential key mechanisms and pathophysiological alterations related to specific risk factors and comorbidities conditioning susceptibility to AF, evolution of the AF burden and progression from paroxysmal to permanent AF^65–67^ are summarized in *Table 4*.

**Table 2:** Determinants of incident AF across large epidemiological population-based studies and impact on risk factors/comorbidities

Study	Population	Study start date	AF incidence	Determinants of incident AF
Multi-Ethnic Study of Atherosclerosis (MESA)^S1–S8^	>6000 patients Aged >45 years 38% Whites, 28% Black race	Start in 2000	Incident rate 5.2 per 1000 person‐years	Maximum LAVI: HR 1.38 Hypertension: HR 2.6 PAD: HR 1.5 Sleep apnoea: HR 1.76 CMR-derived LVM :HR 1.45 CAC >300: HR 2.1
Rotterdam Study^S9,S10^	>10 000 patients Aged >55 years	Start in 1990	Overall incidence 9.9/1000 person-years Lifetime risk 23.8% for men aged 55 and 22.2% for women aged 55 years	Dementia: OR 2.3 Cognitive impairment: OR 1.7
Framingham Heart Study^S11,S12^	>5000 patients Aged >55 years	Start in 1948	Lifetime risk 25.9% for men and 23.2% for women aged 50 years	Diabetes: OR 1.4 for men and 1.6 for women LVH: OR 1.4 for men LVH: OR 1.6 for women Hypertension: OR 1.5 for men and 1.4 for women MI: OR 1.4 for men and 1.2 for women HF: OR 4.5 for men and 5.9 for women Valvular heart disease: OR 1.8 for men Valvular heart disease: OR 3.4 for women
The Atherosclerosis Risk in Communities (ARIC) study^S13,S14^	>15 000 patients >45 years 27% Black race	Start in 1985	Overall incidence 4/1000 person-years in White women, 6.7/1000 person-years in White men; 3/1000 person-years in Black women, 3.9/1000 person-years in Black men	Black race :HR 0.6 Male sex: HR 1.92 BMI (kg/m^2^) ≥30: HR 1.78 SBP ≥160 mmHg: HR 2.63 LVH: HR 2.73 LA enlargement: HR 1.61 Diabetes: HR 1.87 CAD: HR 2.21 HF: HR 3.03 eGFR (mL/min/1.73 m^2^) HR 3.75 for 15–29 mL/min
Cardiovascular Health Study^S15–S17^	>5000 patients Aged >65 years 15.4% Black race	Start in 1988	19.2 per 1000 person-years among adults ≥65 years old	BMI (kg/m^2^, per 5 units): HR 1.1 in Whites, 1.31 in Blacks SBP (per 20 mmHg): HR 1.15 in Whites and Blacks Hypertension: HR 1.55 in Blacks LVM/BSA (per SD): HR 1.21 Diabetes: HR 1.56 in Whites Nt-proBNP (per log-pg/dL): HR 1.63 in Whites and 1.64 in Blacks LA dimension (per 0.5 cm): HR 1.26 in Whites and 1.29 in Blacks
Manitoba Follow-Up Study^S18^	>3900 patients Mean age 31 years	Start in 1948	AF incidence 0.5/1000 person-years <50 years; 16.9/1000 person-years >85 years	MI: RR 3.62 HF: RR 3.37 Hypertension: RR 1.42 Obesity: RR 1.28 Valvular heart disease: RR 3.15

AF, atrial fibrillation; BMI, body mass index; BSA, body surface area; CAC, coronary artery calcium; CAD, coronary artery disease; HF, heart failure; LA, left atrial; LAVI, left atrial volume index; LVH, left ventricular hypertrophy; LVM, left ventricular mass; MI, myocardial infarction; PAD, peripheral artery disease; SBP, systolic blood pressure; SD, standard deviation.

**Table 3:** Impact of comorbidities on AF-associated outcomes, according to real-world registries on AF patients

Registry	Study design	Population	Start date and follow-up	Independent predictors of adverse outcomes	
				Mortality	Other outcomes of interest
ORBIT-AF (I–II)^S19–S26^	Prospective multicentre nationwide registries that enrolled patients with incident and prevalent AF across the USA (>200 sites overall)	ORBIT AF I 10 137 ORBIT AF II 13 404	ORBIT AF I 2009 2 years ORBIT AF II 2013 3 years	SBP ≤120 mmHg: HR 0.86 Diabetes: HR 1.63 (patients <70 years old) Diabetes: HR 1.25 (patients ≥70 years old) Obesity: HR 0.73 Heart failure: HR 1.69	SBP (every 5 mmHg increase) Stroke/SE/TIA: HR 1.05 Myocardial infarction: HR 1.05 Major bleeding: HR 1.03 Diabetes SCD: HR 1.53 All-cause hospitalization: HR 1.15 Heart failure Hospitalization: HR 1.31 Vascular disease MACNE: HR 1.83 CV death: HR 2.16 MI: HR 3.50 OSA MACNE: HR 1.16 Stroke/SE/TIA: HR 1.38 Bleeding hospitalization: HR 1.18
GARFIELD AF^S27–S29^	Observational, prospective, multicentre study of patients with newly diagnosed AF and one or more additional risk factors for stroke from 35 countries worldwide (excluding USA)	57 000	2009 Minimum 2 years, up to 7 years	Diabetes: HR 1.27 Hypertension: HR 0.86 Heart failure: HR 1.86 Vascular disease: HR 1.40 CKD: HR 1.72	Stroke/SE Diabetes: HR1.23 Heart failure: HR 1.33 Vascular disease: HR 1.35 CKD: HR 1.62 Major bleeding Vascular disease: HR 1.39 CKD: HR 1.74
ARAPACIS^S30–S33^	National, multicentre, observational, prospective study enrolling AF out- or in-patients, from 136 centres	2027	2010 3 years	Heart failure: HR 2.02 Vascular disease: HR 1.41 COPD: HR 2.16	MACE Heart failure: HR 1.56 Vascular disease: HR 1.97 COPD: HR 1.77 CKD: HR 2.2 CAD: HR 2.07
ESC-EORP AF General Pilot^S34–S40^	Prospective, multicentre, observational registry held in 9 ESC countries, enrolling consecutive AF patients in 67 cardiology practices	3119	2012 3 years	Heart failure: OR 2.09 Diabetes: OR 1.63 CKD: OR 1.97 No physical activity: OR 2.18	Composite outcome ^a^ Heart failure: OR 2.18 Diabetes: OR 1.67 CKD: OR 2.35 No physical activity: OR 2.71
ESC-EORP AF Long-Term^S41,S42^	Prospective, multicentre, observational registry held in 27 ESC countries, enrolling consecutive AF patients in 250 cardiology practices	11 906	2013 2 years	Heart failure: HR 2.17 Any cardiomyopathy: HR 1.74 PAD: HR 1.36 CKD: HR 1.78	Any TE/ACS/CV death Heart failure: HR 1.79 Diabetes: HR 1.22 PAD: HR 1.29 CKD: HR 1.54 CAD: HR 1.32

ACS, acute coronary syndrome; AF, atrial fibrillation; ARAPACIS, Atrial fibrillation Registry for ABI Prevalence Assessment-Collaborative Italian Study; CKD, chronic kidney disease; CV, cardiovascular; COPD, Chronic obstructive pulmonary disease; ESC, European Society of Cardiology; EORP AF, EURObservational Research Programme on Atrial Fibrillation; GARFIELD-AF, Global Anticoagulant Registry in the FIELD e Atrial Fibrillation; GLORIA-AF, Global Registry on Long-Term Oral Antithrombotic Treatment in Patients with Atrial Fibrillation; HR, hazard ratio; MACE, Major adverse cardiovascular events; MACNE, major adverse cardiac and neurologic event; MI, myocardial infarction; OR, odds ratio; ORBIT-AF, Outcomes Registry for Better Informed Treatment of Atrial Fibrillation II; OSA, obstructive sleep apnea; PAD, peripheral artery disease; SBP, systolic blood pressure; SCD, sudden cardiac death; SE, systemic embolism; TIA, transient ischaemic attack.

a HR for the composite outcome of stroke/TIA/peripheral embolism/all-cause death.

**Table 4:** Potential key mechanisms and pathophysiological alterations related to specific risk factors and comorbidities conditioning susceptibility to AF, evolution of the AF burden and progression from paroxysmal to permanent AF^65–67^

Risk factor or comorbidity	Potential mechanisms and pathophysiological alterations
Heart failure	Atrial structural remodelling Abnormal calcium handling Proinflammatory activation Abnormal neurohumoral and adrenergic activation
Hypertension and LV hypertrophy	Atrial structural remodelling Impaired left diastolic function RAAS activation Conduction slowing
Obesity	Atrial structural remodelling Impaired left diastolic function Increased epicardial adipose tissue
Diabetes mellitus	Atrial structural remodelling Autonomic nervous system imbalance Inflammation and oxidative stress Insulin resistance
Chronic kidney disease	Atrial structural remodelling Inflammation and oxidative stress RAAS activation Changes in calcium and phosphate metabolism
Acute MI and coronary artery disease	Conduction slowing, block Atrial structural remodelling Abnormal calcium handling Left ventricular dysfunction Altered autonomic activity Inflammation and oxidative stress
Peripheral artery disease	Atrial structural remodelling Inflammation and oxidative stress Endothelial damage/dysfunction
Obstructive sleep apnoea	Atrial structural remodelling Conduction slowing Sympathetic activity induced by hypoxia Autonomic nervous system imbalance Fluctuation of intrathoracic pressure (left atrial overload) Inflammation and oxidative stress

LV, left ventricular; MI, myocardial infarction; RAAS, renin–angiotensin–aldosterone system.

### 4.1 Heart failure and ventricular dysfunction

#### 4.1.1 Heart failure as a factor facilitating AF onset

HF and AF represent two major health problems often weaved in a reciprocal relationship in which one favours both onset and maintenance of the other. HF has been associated with a more than threefold risk of incident AF,^68^ with an overall prevalence of AF ranging from 27% in HF with reduced ejection fraction (HFrEF) to 39% in HF with preserved ejection fraction (HFpEF) in the ESC HF long-term registry.^69^ The reciprocal relationship between AF and HF is confirmed by data indicating that AF was reported in 57% of patients with new HF diagnosis^70^ and almost one-third of those with chronic HF.^71^

#### 4.1.2 Heart failure and AF burden

Large HF trials have revealed that the likelihood of AF increases with the severity of HF, ranging from 10% in HF with New York Heart Association (NYHA) class I to class II symptoms to 50% in HF with NYHA class IV symptoms.^72^ HF was an independent predictor of clinical AF progression, estimated as 5.2 per 100 patient-years in the 3-year follow-up of the Swiss AF study.^73^ While many studies were focused on HF with systolic dysfunction, the prevalence of AF is even higher in HFpEF.^74^ In many studies on HF, persistent or permanent AF is more common than paroxysmal AF, indicating that HF is more frequently associated with more advanced types of AF. Among the 15 415 patients randomized in the ATMOSPHERE and PARADIGM trials, 35.6% patients had a history of AF and two-thirds of these had persistent or permanent AF, with paroxysmal AF accounting for one-third of the cases.^75^ In observational studies, long-standing persistent or permanent AF is the most commonly diagnosed type of AF, accounting for 40–50% of cases, with 20–30% of patients presenting paroxysmal and 20–30% presenting persistent AF.^74^ Most of the available data regarding the relationship between HF and AF are based on studies of clinical AF (i.e. 12-leads ECG confirmed AF). In a sub-analysis of the ASSERT study, even the progression of subclinical AF (i.e. progression of subclinical AF to episodes >24 h), was associated with a five-fold increase in the risk of HF hospitalization suggesting that AF burden may also modulate outcomes in HF patients.^76^

#### 4.1.3 Heart failure as a factor modulating the outcome of AF patients

Either HF and AF are related to adverse patient outcomes, in terms of hospitalization rates, and increased overall mortality. Co-existing HF and AF lead to even poorer outcomes and increased mortality than either condition alone, as well as increased risk of stroke and TE.^77^^,^^78^ Nonetheless, among patients who present with both diseases implications on outcomes are not homogeneous, mainly in relation to temporal sequence of onset and different HF subtypes (preserved vs. reduced ejection fraction).^77^ In the Framingham study, patients with HF had around a doubled risk of incident AF compared to patients free of HF, even after adjustment for age and sex, with a slightly higher risk of developing AF in subjects with pre-existing HF compared to patients who developed HF along with time, before AF.^70^ The incidence rates of mortality in AF patients were highest in participants who had HFrEF at baseline, followed by those with previous HFpEF and lowest in those without HF.^70^ As an independent prothrombotic condition, HF is encountered in most common risk scores (CHADS_2_, CHA_2_DS_2_-VASc, ATRIA) for TE risk assessment in AF and carries an increasing yearly incidence of stroke by 0.054% for each percentage point of ejection fraction decrease (95% CI 0.013–0.096%).^79^ However, in patients with AF, no differences between HFpEF and HFrEF had been observed in thromboembolic risk.^80^

#### 4.1.4 Translational implications

The frequent coexistence of HF and AF raises the question of which comes first, and which pathways lead from a disease to the development of the other (‘AF begets HF and vice versa’).^70^ The sharing of common risk factors, such as age, hypertension, diabetes, ischaemic-, and valvular heart diseases only partially explains this relation, since each condition may directly promote the other. Chronically, HF causes interstitial fibrosis and conduction abnormalities,^81^ promotes increased atrial filling pressures with atrial dysfunction and local remodelling, and sustains abnormal automaticity and triggered activity through neurohumoral and adrenergic activation,^82^ all these mechanisms providing a favourable substrate for AF onset. In this situation, patients with first diagnosed HF should be regularly monitored to detect AF. In case of first detected AF, a strategy of early rhythm-control that includes AF ablation could be associated with better outcomes and could be a good approach for slowing down the vicious circle between HF and AF.^83^

### 4.2 Hypertrophy and hypertension

#### 4.2.1 LV hypertrophy and hypertension as factors facilitating AF onset

Hypertension is one of the most common cardiovascular disorders, currently affecting up to 50% of the global adult population.^84^ In population studies, hypertension is an independent predictor for incident AF, carrying an almost doubled risk in Framingham Heart Study and 1.4-fold increased risk in Manitoba Follow-up Study.^85^^,^^86^ Due to its higher prevalence, elevated blood pressure (BP) is the single risk factor accounting for more AF cases than any other, explaining more than one-fifth of all new AF cases in the ARIC (Atherosclerosis Risk in Communities) study cohort^87^ and reaching an overall prevalence up to 90% among AF population of clinical trials. To date, BP is related to the risk of incident AF by a U-shaped relation, in which the lowest risk for new AF may be obtained through BP levels <130/80 mmHg in patients under 80 compared to less severe thresholds in those aged >80.^88^ Being an end-product of the hypertensive state, left ventricular (LV) hypertrophy (LVH) has also been identified as a predictor of new AF in many registries.^89^ Although the prevalence of LVH in the hypertensive population broadly varies among studies according to different diagnostic criteria adopted, AF prevalence seems to be higher in patients with more severe ventricular remodelling (especially in the case of concentric and eccentric hypertrophy).^90^ LVH detected by ECG criteria predicts AF independently of LV mass assessed by echocardiography or magnetic resonance.^91^ Moreover, the presence and severity of LVH, assessed by Cornell product, predicted the new-onset of AF in the Losartan Intervention For End Point Reduction in Hypertension (LIFE) study (HR 1.01, 95% CI 1.00–1.02),^92^ while its regression was associated with a decreased risk of incident AF (for every 1 standard deviation lower Cornell product, adjusted HR 0.88; 95% CI 0.80–0.97).^93^

#### 4.2.2 LV hypertrophy, hypertension, and AF burden

Among recently diagnosed AF patients included in the RecordAF cohort, hypertension carried a 1.5-fold increased risk of AF progression after a 1-year follow-up (OR 1.5, 95% CI 1.1–2.0), and accounted for even greater risk in the subgroup selected for a rhythm-control strategy (OR 1.8, 95% CI 1.2–2.7).^94^ The higher progression rate observed among hypertensive patients could be mainly explained by the presence of LVH, which conferred an almost five-fold increased risk of AF progression among the 799 patients from the Euro Heart Survey after 1-year follow-up (OR 4.84, 95% CI 1.70–13.78). However, the independent effect of LVH on AF progression was found only among male patients, while in hypertensive female subjects the progression rates in patients with and without LVH were similar.^95^ Since previous studies have shown gender-specific differences in LV remodelling and taking into account that men have predominantly concentric hypertrophy, these findings may suggest that the effect of LVH on AF progression is not homogeneous and may be related to the type of hypertrophy, as well as other factors.^96^

#### 4.2.3 LV hypertrophy and hypertension as factors modulating the outcome of AF patients

Hypertension is a major, independent risk factor for stroke, incremental with coexisting AF. The close relation between BP and cerebrovascular disease/stroke risk accounts for its relevance in common thromboembolic risk scores (CHADS_2_, CHA_2_DS_2_VASc, ATRIA, ABC, Garfield).^1^ Mean systolic BP levels above 140 mmHg significantly increased the thromboembolic events rates among the anticoagulated AF population of the SPORTIF trials (HR 1.83, 95% CI 1.22–2.74).^97^ Similar data from the ARISTOTLE trial population showed that high BP was associated with an increased risk for stroke (HR 1.53, 95% CI 1.25–1.86)^98^ and in a substudy from the ROCKET-AF trial, uncontrolled hypertension carried an even higher stroke risk compared to controlled BP levels (HR 1.42, 95% CI 1.03–1.95).^99^ Hypertension has also an impact on microvascular disease in patients with AF. Various types of brain lesions, such as white matter lesions, have been observed in unselected cohorts of hypertensive AF patients with important clinical implications (e.g. increased risk of stroke, cognitive impairment and vascular dementia).^100^

#### 4.2.4 Translational implications

The cornerstone of AF pathophysiology in both hypertension and LVH is the extent of cardiac remodelling. Besides the epidemiological associations and well-established structural changes, the pathogenic pathways leading from hypertension to AF are still not completely explored.^101^ Long-standing hypertension promotes haemodynamic changes leading to increased LV thickness and stiffness, early impairment of LV diastolic function and subsequent atrial dysfunction and progressive enlargement, finally predisposing to AF.^102^ Histological changes of cardiac remodelling, both in ventricular and atrial myocardium include alterations of extracellular matrix, fibroblasts proliferation, and myocyte hypertrophy.^103^

### 4.3 Obesity and BMI

#### 4.3.1 Obesity as a factor facilitating AF onset

Increasing body mass index (BMI) is an independent risk factor for AF occurrence.^104^ In a meta-analysis including 123 249 patients, the presence of obesity was associated with an increased risk of AF (risk ratio 1.08).^105^ In a more recent meta-analysis, which investigated the relationship between continuous BMI and the risk for AF, the authors showed that an increase in five BMI units was associated with a 28% increase in the relative risk of presenting AF with a non-linear relationship.^106^

#### 4.3.2 Obesity and AF burden

Beyond being a risk factor for AF occurrence, increasing BMI has also been found associated with a higher burden of AF.^107^ In a subgroup analysis of the AFFIRM trial, being overweight and obesity were associated with a higher burden of AF in the rate control arm, and with an increased use of cardioversion procedures in the rhythm control arm.^107^ In a longitudinal cohort study, BMI independently predicted progression to permanent AF and both being overweight and obesity were independently associated with progression to permanent AF.^108^

#### 4.3.3 BMI as a factor modulating outcomes in AF patients

Several studies have investigated the relationship between obesity or being overweight and the occurrence of major adverse events in AF patients.^109^^,^^110^ In the last few years, a phenomenon has emerged related to clinical research in the field of obesity studies, the so-called ‘obesity paradox’.^111^ The latter is related to obese patients having a lower risk of short-term and long-term adverse outcomes.^111^ While the large majority of studies deriving from randomized controlled trials (RCTs) subgroup analyses have shown an independent association with a risk reduction for stroke, cardiovascular death and all-cause death outcomes for overweight and obese patients, most observational and population-based cohort studies, in which the obesity paradox is hypothesized, have reported controversial results.^104^ In a systematic review and meta-analysis investigating the issue of the obesity paradox in AF patients,^112^ the risk for adverse outcomes in overweight and obese patients with AF is substantially similar to the risk of AF patients of normal weight.^112^ This topic remains controversial since these conclusions are challenged by other two meta-analyses, pooling together data derived from RCTs, showing that patients with a higher BMI have a lower risk for stroke occurrence.^113^

#### 4.3.4 Translational implications

Several mechanisms have been suggested to explain the relationship between obesity and AF. Obese patients have been found to present a number of modifications in physiology and morphology of the heart that can directly cause occurrence of HF and AF.^114^ Furthermore, the presence of increased epicardial adipose tissue is able to induce a paracrine pro-inflammatory status (with increased interleukin-1 beta and tumour necrosis factor alpha) that, beyond the aforementioned cardiac structural changes, may lead to AF onset.^115^

### 4.4 Diabetes mellitus

#### 4.4.1 Diabetes as a factor facilitating AF onset

Diabetes mellitus (DM) is an independent risk factor for the onset of AF, as initially shown in the Framingham Heart Study.^68^ In a 38-year follow-up, the study found that the development of AF was independently associated with diabetes with an OR of 1.4 for men and 1.6 for women. The ARIC study found that a diagnosis of DM, poor glycaemic control in diabetic patients and HbA1c levels, the latter both in diabetic and non-diabetic subjects, were independently associated with an increased risk of AF.^116^ In a meta-analysis based on seven prospective cohort studies and four case-control studies, including more than 108 000 cases of AF, patients with DM had an approximate 40% greater risk of AF compared to unaffected patients (relative risk 1.39).^117^ However, additional investigations are probably needed, since in a recent population study, with a 12.6-year median follow-up, the association between diabetes and the population attributable risk of incident AF was not confirmed, for both sexes.^118^

#### 4.4.2 Diabetes and AF burden

Patients with diabetes have a higher burden of AF since they present more frequently with permanent AF, as observed both in the EORP-AF registry^119^ and the ORBIT AF registry.^120^

#### 4.4.3 Diabetes as a factor modulating the outcome of AF patients

In real-world registries up to 20% of AF patients have DM.^119^ Diabetes is a known risk factor for TE and stroke events in patients with AF, associated with a 70% relative increase in risk of stroke.^121^ In patients aged less than 70 years old enrolled in the ORBIT AF Registry,^120^ diabetes was associated with a 63% increase in total mortality at a 2-year follow-up, and with a 120% increase in cardiovascular mortality. Patients with AF and diabetes also had a higher incidence of sudden cardiac death, hospitalizations, and cardiovascular hospitalizations.^120^

#### 4.4.4 Translational implications

*S*everal possible pathways may be involved in conditioning a milieu favouring AF onset, through an effect on action potential duration of myocytes and associated electrical and structural remodelling.^122^

The role of hyperglycaemia associated with DM and insulin resistance, which is usually related also to hypertension and obesity, has been assessed in experimental studies. However, nowadays, the use of continuous glucose monitoring systems may offer detailed information on the accuracy and variability in glycaemic control in diabetic patients and may allow us to investigate the interaction between AF and glucose fluctuations.^123^ This can lead to specific therapeutic interventions targeted to prevent AF and reduce its burden in diabetic patients.

### 4.5 Chronic kidney disease

#### 4.5.1 CKD as a factor facilitating AF onset

AF and chronic kidney disease (CKD) have a bi-directional link and the presence of CKD increases the risk of incident AF, while the presence of AF is associated with the progression of CKD.^124^ In the Chronic Renal Insufficiency Cohort (CRIC) study, individuals with estimated glomerular filtration rate (eGFR) <45 mL/min/1.73 m^2^ had a higher prevalence of AF compared with participants with eGFR ≥45 mL/min/1.73 m^2^ (20.4% vs. 16.0%; *P* = 0.001).^125^ Of note, clinical factors known to be predictors of AF in the general population (such as race/ethnicity, hypertension, diabetes, BMI, physical activity, total cholesterol, and alcohol intake) were not significantly associated with AF in CKD patients.^125^ These findings highlight the unique pathophysiological link between AF and CKD, suggesting that AF risk prediction models developed in the general population may not be sufficiently valid in CKD cohorts. In a large population-based cohort study conducted in Sweden, enrolling 116 184 individuals with CKD, an eGFR <30 was associated with a 1.6-fold increased risk of incident AF.^126^ Similarly, a prospective community-based observational cohort study including 235 818 subjects based upon a voluntary annual health check-up program in Japan, found that the risk of incident AF was higher with decreasing GFR.^127^

#### 4.5.2 CKD and AF burden

In observational studies, AF patients show a completely different pattern of AF types comparing patients with different degrees of altered renal function, with a lower prevalence of paroxysmal AF and a higher prevalence of permanent AF with eGFR below 50 or, even greater, with eGFR below 30 mL/min/1.73 m^2^.^128^ A recent meta-analysis found that CKD patients presented more AF recurrences 30 days after electrical cardioversion (OR 2.62, 95% CI 1.28–5.34) and a higher incidence of AF recurrences at long-term after catheter ablation (HR 1.69, 95% CI 1.22–2.33).^129^

These findings may suggest an association between AF burden and more advanced CKD, but the interpretation of this relationship in a cause–effect rapport is unclear given the presence of other comorbidities, such as hypertension.^128^^,^^130^

#### 4.5.3 CKD as a factor modulating the outcome of AF patients

Overall, AF patients with CKD have a significantly higher risk of adverse outcomes, including all-cause mortality, compared to those without CKD.^128^^,^^131^ Independently of AF, CKD is a pro-thrombotic and pro-haemorrhagic condition and it is not surprising that AF patients with concomitant CKD are at a high risk of stroke, TE and major bleeding.^130^ As highlighted in the meta-analysis by Providência *et al*.,^131^ the presence of CKD in patients with AF is associated with an almost 50% increase in thromboembolic risk (HR 1.46, 95% CI 1.20–1.76) especially with end-stage CKD (HR 1.83, 95% CI 1.56–2.14). Despite the essential evaluation of renal function in AF patients, as CKD is associated with a poor overall prognosis in terms of TE events and all-cause mortality, the addition of renal impairment to the CHADS_2_ or CHA_2_DS_2_VASc seems to not improve the predictive value of these scores.^124^^,^^132^ Indeed, since renal impairment is associated with all stroke risk factors listed within the CHADS_2_ and CHA_2_DS_2_VASc, it does not have an independent additive predictive value.^133^

Renal impairment also increases the risk of bleeding. Several pathophysiological mechanisms have been proposed such as haemostatic defects, platelet dysfunction and altered platelet-vessel wall-interaction etc.^124^^,^^130^

#### 4.5.4 Translational implications

Several mechanisms have been proposed to account for why AF is more common in CKD patients.^130^ CKD is associated with many arrhythmogenic substrates, which can result in the development of AF.^124^ Patients with advanced stage of CKD usually have a higher burden of AF due to different potential mechanisms, such as activation of the renin–angiotensin–aldosterone system (RAAS), atrial remodelling, elevated levels of inflammatory markers, augmented sympathetic tone, and many other factors which are not yet completely elucidated.^130^ The activation of the RAAS seems to be one of the most important links between AF and CKD.^130^ RAAS activation indeed increases the production of reactive oxygen species and is involved in several processes such as the up-regulation of cytokines, profibrotic growth factors, extracellular matrix proteins which can promote atrial fibrosis and structural remodelling.^130^^,^^134^ Among these elements, transforming growth factor-β1 (TGFβ1) has a central role in fibrogenesis and is one of the key elements of atrial structural remodelling. However, the exact mechanisms involved in the development of atrial fibrosis are unknown. A recent study investigated the potential pathogenesis of AF in a rat model finding that CKD (experimentally produced by nephrectomy) led to LA enlargement, increased the vulnerability to AF with abnormal P-waves.^135^ The authors interestingly found that the marked up-regulation of the TGFβ1 pathway in CKD rats produced severe interstitial fibrosis by a massive extracellular matrix deposition of collagen type I and α-smooth muscle actin.^135^ Moreover, oxidative stress may be involved in the pathogenesis of LA fibrosis and enhanced AF vulnerability in experimental models of CKD.^136^

### 4.6 Acute myocardial ischaemia and coronary artery disease

#### 4.6.1 Ischaemia as a factor facilitating AF onset

While the prevalence of AF among coronary artery disease (CAD) populations seems relatively low, ranging between 0.2% and 5%, the prevalence of CAD among AF patients is significantly high (between 13% and 46%).^137^ Notably, both diseases are promoted by inflammation and share many risk factors: hypertension, DM, sleep apnoea, obesity, and smoking.^138^ Despite many authors reported an association between atherosclerosis and AF, we have limited data confirming this hypothesis in prospective cohorts of patients without clinical manifestations of AF or CAD.^139^ Two large registries show a close relationship between CT assessed calcium score and later occurrence of AF, while an analysis from the Danish registry evidenced a correlation with baseline calcium score (area under the curve 0.68) especially for values ≥1000.^140^ In the setting of acute coronary syndromes, acute myocardial infarction (MI) is an established risk factor for AF development, occurring in 6–21% of the patients^141^ with similar occurrence in the pre-thrombolytic and post-thrombolytic era^142^^,^^143^ and mainly associated with patients’ characteristics (e.g. age, comorbidities) and presence/severity of LV dysfunction.^141^ Improvements in clinical outcomes of MI in the recent era have positively affected post-MI incidence of AF.^144^

#### 4.6.2 Ischaemic heart disease and AF burden

Even if AF during acute MI may appear to be a transient AF, long-term follow-up data show an important rate of AF recurrence.^145^ These data suggest that acute AF may indeed result from a complex interaction between ischaemia, as a precipitating event, and an underlying substrate favouring AF. In the RACE V study, evaluating temporal patterns and short-term progression of paroxysmal AF, patients with long AF episodes (>12 h) were more frequently affected by CAD and HF, and patients with higher AF burden (>2.5%) were older and had a higher calcium score supporting an association between CAD and AF temporal profile.^30^

#### 4.6.3 Ischaemic heart disease as a factor modulating the outcome of AF patients

The presence of vascular disease (V) is included in the CHA_2_DS_2_-VASc score, defined as previous MI, peripheral arterial disease (PAD), or complex aortic plaque.^146^ A recent analysis including all patients undergoing coronary angiography registered in the Western Denmark Heart Registry, evidenced that the presence of significant CAD (defined as obstructive coronary stenosis in at least one coronary vessel, or non-obstructive coronary stenoses in ≥2 coronary vessels), but not the number of diseased vessels, was associated with increased risk of the combined endpoint of ischaemic stroke/TIA and systemic embolism.^147^ CAD *per se* is associated with an increased risk of stroke and other cardiovascular events also in patients without AF.^148^ In a sub-analysis of the GARFIELD registry^149^ history of acute coronary syndromes was associated with worse 2-year outcomes, including stroke and mortality, coupled with under-treatment with OACs and wider use of antiplatelet agents. Major bleeding was also more common.

#### 4.6.4 Translational implications

Several mechanisms have been advocated in the promotion of AF by ischaemic heart disease. The easiest conclusion is that the presence of shared similar risk factors and the increased prevalence with age, indeed, the concomitant occurrence of CAD and AF is only driven by modest statistical associations. However, there are several experimental studies demonstrating this association. Acute atrial ischaemia creates a substrate for AF maintenance within several hours, leading to decreased conduction velocity and increased conduction heterogeneity and shortening of atrial effective refractory period.^150^^,^^151^ On the other hand, a higher AF burden could potentially promote intermittent atrial and ventricular ischaemia favouring AF progression. Future studies implementing long-term AF monitoring in specifically designed experimental models could provide new insights on this topic.

### 4.6 Peripheral arterial disease

#### 4.6.1 PAD as a factor facilitating AF onset

PAD, is one of the main manifestations of systemic atherosclerosis with an increasing prevalence and incidence.^152^ Evaluation of Ankle-Brachial Index (ABI) represents the main tool for primary PAD diagnosis,^153^ with an ABI ≤0.90 being diagnostic for the presence of PAD. Beyond sharing similar epidemiology and risk factors, PAD and AF are closely related.^154^ Several studies reported that patients with PAD showed an increasing risk for developing AF, with approximately a 30% increase in risk for PAD vs. non-PAD subjects.^154^^,^^155^

#### 4.6.2 PAD and AF burden

There are no studies reporting specific data on the relationship between PAD and differential AF burden. In a study reporting data about asymptomatic PAD in AF patients, an intima-media thickness higher than 0.90 mm, indicating subclinical atherosclerosis, was found more frequently associated with persistent/permanent AF, rather than with paroxysmal AF.^156^

#### 4.6.3 PAD as a factor modulating occurrence of outcomes in AF patients

The presence of symptomatic PAD is clearly recognized as a major risk factor for stroke in AF patients, also being part of CHA_2_DS_2_-VASc score.^5^ Furthermore, PAD was found associated with an increasing risk for several major adverse events in AF patients.^155^ In particular, AF patients with symptomatic PAD showed an increased risk for cardiovascular and all-cause death.^155^^,^^157^ Even the presence of asymptomatic PAD was found associated with an increased risk of adverse events with an ABI ≤0.90 associated with the occurrence of vascular events, MI, and vascular death.

#### 4.6.4 Translational implications

The relation between PAD and AF, particularly the inverse association between ABI and incident AF, underlines a significant possible pathophysiological process. If the occurrence of PAD is due to the presence of multiple risk factors leading to atherosclerosis, we can consider that the presence of atherosclerosis, together with the persistence of risk factors, can perpetuate the inflammatory burden and the endothelial function impairment which can ultimately bring to the occurrence of AF.^154^^,^^158^

### 4.7 Obstructive sleep apnoea

Despite an average prevalence of 10% among middle-aged and older subjects (ranging between 3% and 5% in females and 10–17% in males), up to 24 million adults in the USA remain undiagnosed.^159^

#### 4.7.1 OSA as a factor facilitating AF onset

The strong association between AF and obstructive sleep apnoea (OSA) has been initially shown by the Sleep Heart Health Study (SHHS) trial, a multicentre prospective study on 6441 participants aged ≥40 years.^160^ The authors showed a prevalence of AF of 4.8% among patients with sleep-disordered breathing vs. 0.9% in the remaining cohort (*P* = 0.003).^160^ The severity of OSA was associated with a parallel increase in AF risk^160^ and a temporal association between apnoeic events and AF recurrences has been reported.^161^ The results by the SHHS study^160^ was in line with previous findings reported by Hoffstein and Mateika^162^ among 458 patients undergoing polysomnography showing that patients with an apnoea/hypopnea index (AHI) of ≥10/h had an AF prevalence rate of 58% compared with 42% in those with an AHI of ≤10/h (*P* < 0.0001), and the frequency of AF was even higher (70%) in patients with severe OSA (AHI ≥40/h). Beyond the risk of AF, OSA has been associated with an increased risk of stroke, but data from a US population-based case-control study showed that patients with OSA who experienced a stroke had a significantly higher rate of AF.^163^

#### 4.7.2 OSA and AF burden

Despite the abundance of evidence on the role of OSA in the promotion of AF, we are currently lacking rigorous studies on the effect of OSA on AF progression, while the available data seem discordant. Non-randomized studies have shown an association of OSA with an increase in AF relapses during antiarrhythmic therapy and after electrical cardioversion or radiofrequency catheter ablation.^164^ Moreover, there is a positive effect of continuous positive airway pressure (CPAP) on maintenance of sinus rhythm in general and considering specific interventions.^164^^,^^165^

For example, Full *et al*.^166^ recently reported a prospective observational community-based study on 2306 adults aged 45–64 years assessed for daytime sleepiness and occurrence/burden of AF and other arrhythmias, by a continuous 14 days patch-driven monitoring. Excessive daytime sleepiness self-reported by the patients was not associated with objective measures of arrhythmia burden.^166^ The reasons for these discrepancies can derive from the difficulty in obtaining correct identification of OSA and other sleep disorders in AF patients, and the issues associated with the compliance with CPAP. For these reasons, this topic still deserves the development of properly designed study.

#### 4.7.3 OSA as a factor modulating the outcome of AF patients

OSA and AF are, independently from each other, predictors of worse outcomes (e.g. mortality, stroke and hospitalization) in the general population and among several subgroups of patients with cardiovascular disease. The available data evidence that the clinical risk profile of AF patients with vs. without OSA is worse.^167^^,^^168^ However, the main impact of OSA on AF patients is related to an increased risk of stroke, especially for otherwise low-risk patients and not in terms of mortality or HF events.^168^^,^^169^ If this phenomenon derives from an additive effect, independently increasing these events, or an effect mediated by potentiation of AF deserves additional research.

Notably, the results of the Sleep Apnea Cardiovascular Endpoints (SAVE) study failed to show positive effect of CPAP on the reduction in the composite endpoint of death from any cardiovascular cause, MI (including silent MI), stroke, or hospitalization for HF, acute coronary syndrome (including unstable angina), or TIA^170^ reducing the causative relationship between OSA and AF outcomes at least in patients with established cardiovascular disease.

#### 4.7.4 Translational implications

Beyond the number of risk factors and comorbidities shared between AF and OSA, that can explain their association in many subjects (e.g. driven by obesity) there are many potential mechanisms by which OSA might contribute to promote AF: (i) intermittent hypoxia, (ii) recurrent arousals, (iii) increased negative intrathoracic pressure all inducing an increased sympathetic activity, oxidative stress, endothelial dysfunction, electrical/mechanical remodelling of both atria induced by pre/post-load modifications.^171^ Beyond these explanations, other theories have been recently formulated, like the effects of OSA on the expression of cardiac connexins,^172^ promotion of inflammation and metabolic syndrome.^171^

## 5. AF, multimorbidity and frailty: related scores, clinical implications, and translational perspectives

The increasing prevalence of AF with age obviously faces off against older people affected by multiple comorbidities. Comorbid conditions can be measured using specific scores, such as Charlson Comorbidity Index (CCI)^173^ and Elixhauser’s Comorbidity Measure (ECM)^174^ that give an estimate of the overall burden of comorbidities.

### 5.1 Charlson comorbidity index and AF

The relationship between CCI and AF is not fully elucidated, but since several comorbidities included in CCI may independently promote AF, it is logical to expect that CCI is higher in patients with AF vs. non-AF. This association was confirmed in a large study analysing administrative data where a higher CCI was found in patients with AF vs. patients without AF (1.8 ± 2.1 vs. 0.2 ± 0.9; *P* < 0.001).^175^ Moreover CCI progressively increased over time in patients with and without AF, but in patients with AF was steadily higher compared to those without AF (*P* < 0.001).^175^ Notably, AF patients with higher CCI (≥4) had also higher all-cause mortality, stroke and major bleeding (log-rank *P* < 0.001 for each outcome).^175^ Considering CCI as a continuous variable, any increasing point of CCI was significantly associated with risk of stroke (HR 1.04 95% CI 1.03–1.06), major bleeding (HR 1.03, 95% CI 1.01–1.06) and all-cause mortality (HR 1.10, 95% CI 1.09–1.11).^175^

### 5.2 Elixhauser’s comorbidity measure and AF

ECM is an index of comorbidity proposed to evaluate outcome in hospitalized patients using analysis of administrative data taking into account 30 comorbidities (17 common of CCI plus other 13 not included in CCI).^174^ Some differences have to be highlighted. ECM also includes psychologic/psychiatric categories but does not consider dementia that is crucial and frequently observed in patients with AF.^176^ Moreover, compared to CCI, there are less data regarding the relationship between ECM and AF. One recent study found that ECM, together with cognitive impairment (Mini Mental State Exam adjusted for age and educational status < 24) is an independent predictor of outcomes (all-cause mortality and a composite of mortality, TE and bleeding, new or worsening of HF) in a cohort of patients with AF.^177^

#### 5.3 Frailty and AF

Beyond the mere concept of multimorbidity, the paradigm of frailty corresponds to a more complex medical syndrome, of which multimorbidity is only one dimension. Frailty is characterized by a reduced physiologic reserve, which makes subjects more vulnerable to stressors.^178^ In recent years, the concept of frailty has been investigated in relation to cardiovascular diseases.^179^ Although there is wide variability (from 4% to 75%), the prevalence of AF has been reported to be substantial in frail patients.^180^ Nevertheless, it is currently unclear if the presence of frailty can significantly affect AF management.^181^ Beyond the impact on OAC use, several studies have reported that frail AF patients have an increased risk for all-cause death, while it still unclear the impact on stroke and bleeding risk.^182^ Observational data suggest that compliance with the simple ABC pathway is associated with improved outcomes in AF patients with high frailty risk, supporting the value of integrated AF management also in frail subjects.^183^

Further data are still needed to fully elucidate the epidemiology and impact of frailty in AF patients.

#### 5.4 Translational implications

The diagnosis of comorbidities is traditionally based on a clinical approach, and scores for comorbidities are therefore based on diagnosed diseases. Whether a clinical diagnosis of comorbid diseases could be replaced by a set of biomarkers (including e.g. AF burden) is yet uncertain but would enhance a more objective and quantifiable assessment of comorbid diseases and related pathophysiological alterations. The set would include hormones, metabolomics, proteomics, genomics and epigenetics among other dynamic markers of tissue injury.^184^ Once available, it can promote knowledge on the specific pathways of diseases and enhance the selection of the most robust biomarkers, and certainly individualize patient characterization. How AF burden would perform in this context should be researched, but unfortunately current large biobanks lack information on continuously monitored AF patients.^185^

## 6. Towards the future

In the future, there is a need for specific and updated pathways to employ risk factors as clinical tools for assessing AF susceptibility, in terms of risk of incident AF, and also targeting initiatives for AF screening.^13^^,^^40^^,^^186^^,^^187^ Furthermore, for potential prediction of the transition from a low to a high AF burden, as well as from paroxysmal to persistent or permanent forms of AF. Definition of the specific role of comorbidities and risk factors in modulating the complex pathophysiological processes of AF, also in the perspective of potential ‘upstream therapies’ targeted to act on the factors involved in remodelling, still constitutes a gap of knowledge.^66^ A detailed analysis of the pathophysiology of AF according to experimental models is beyond the aim of this review, and we refer to other sources.^188^ However, there are several translational implications coming from basic science. For instance, micro-RNA, i.e. small non-coding RNA regulating target genes, have an expression profile that may change in response to pathological conditions and comorbidities and contribute to the atrial remodelling process.^66^ The accumulation of clinical factors increases the susceptibility to AF, but the specific markers for predicting AF onset and burden increase, according to specific substrates (HF, CKD, OSAS, etc.) or risk factors (diabetes, hypertension), have yet to be precisely identified.^189^ Knowledge of AF mechanisms and susceptibility for well-defined patient subsets, defined on the basis of traditional aetiologies or on the basis of clustering according to analysis of large datasets, can be the basis for new approaches to counteract AF susceptibility and progression at an individual level.^189^ The precise measurement of AF burden may allow for better patient characterization with the possibility of intervention when critical thresholds of burden are attained. It is clear that risk factors and comorbidities have a complex interplay with genetic factors and several molecular determinants of electrogenesis and remodelling, but the molecular pathways involved in these processes are in the early stage of identification.^66^^,^^188^ From this perspective, translational research has great potential for improving our knowledge and guiding the therapeutic approaches to AF.^188^ Initiation and perpetuation of AF have been a matter of investigation at the experimental level, but the factors associated with the transition from the stage of susceptibility to AF, with a limited AF burden, to the stage of permanent AF are still the subject of investigation with the potential for important translational implications. Our knowledge of the dynamic changes in AF patterns in humans could benefit from the increasing introduction of wearables and apps in practice and clinical research, with the potential for analysis and interpretation of such data, as well as the use of machine learning and artificial intelligence, in line with the perspectives of an advanced use of digital health care.^39^ Wearables and devices for remote monitoring have been traditionally proposed for AF detection, and for detecting signals that can help predict worsening cardiac function. A new perspective is to use these digital tools for assessing the severity of AF burden and its temporal changes, in an attempt to capture the dynamic aspects of AF susceptibility.^40^^,^^190–192^ Notably, such improved methods for data analysis are based on large amounts of data derived from continuous monitoring of AF (through wearables) and could be the basis for assessing how basic knowledge on thrombogenesis and atrial remodelling have an impact on outcomes in the clinical setting. This approach should include both translation from basic science to the clinical setting as well as ‘backward translation’ from clinical to basic science. In view of the complex relationships between AF susceptibility, AF burden and AF evolution, the analysis of the relationships with cardiac substrate and comorbidities should be approached in a multidimensional view, taking into account the complexity of analysing cause and effect relationships, thus suggesting to explore the use of approaches based on chaos theories, as already proposed for complex adaptive systems.^193^ Attempts to improve knowledge and improve event prediction could also benefit from new approaches targeted to better weight the role of the multiple factors conditioning the outcomes of AF patients and by better defining AF phenotypes through cluster analysis.^194^

## Supplementary material

Supplementary material is available at *Cardiovascular Research* online.

**Conflict of interest:** GB: small speaker fee from Medtronic, Boston, Boehringer Ingelheim and Bayer. GYHL: Consultant and speaker for Bayer/Janssen, BMS/Pfizer, Boehringer Ingelheim, and Daiichi-Sankyo (No fees are directly received personally). All the disclosures happened outside the submitted work. Other authors have no disclosures to declare.

## Supplementary Material

cvab147_Supplementary_Material

## References

[1] Lip GYH , Banerjee A, Boriani G, Chiang CE, Fargo R, Freedman B, Lane DA, Ruff CT, Turakhia M, Werring D, Patel S, Moores L. Antithrombotic therapy for atrial fibrillation: CHEST guideline and expert panel report. Chest 2018;154:1121–1201. 30144419 10.1016/j.chest.2018.07.040

[2] Boriani G , Proietti M, Laroche C, Fauchier L, Marin F, Nabauer M, Potpara T, Dan GA, Kalarus Z, Diemberger I, Tavazzi L, Maggioni AP, Lip GYH; EORP-AF Long-Term General Registry Investigators; Steering Committee (National Coordinators). Contemporary stroke prevention strategies in 11 096 European patients with atrial fibrillation: a report from the EURObservational Research Programme on Atrial Fibrillation (EORP-AF) Long-Term General Registry. Europace 2018;20:747–757. 29016832 10.1093/europace/eux301

[3] Proietti M , Lip GYH, Laroche C, Fauchier L, Marin F, Nabauer M, Potpara T, Dan GA, Kalarus Z, Tavazzi L, Maggioni AP, Boriani G; ESC-EORP Atrial Fibrillation General Long-Term Registry Investigators Group. Relation of outcomes to ABC (Atrial Fibrillation Better Care) pathway adherent care in European patients with atrial fibrillation: an analysis from the ESC-EHRA EORP Atrial Fibrillation General Long-Term (AFGen LT) Registry. Europace 2021;23:174–183. 33006613 10.1093/europace/euaa274

[4] Vitolo M , Proietti M, Harrison S, Lane DA, Potpara TS, Boriani G, Lip GYH. The Euro Heart Survey and EURObservational Research Programme (EORP) in atrial fibrillation registries: contribution to epidemiology, clinical management and therapy of atrial fibrillation patients over the last 20 years. Intern Emerg Med 2020;15:1183–1192. 32557091 10.1007/s11739-020-02405-0

[5] Hindricks G , Potpara T, Dagres N, Arbelo E, Bax JJ, Blomström-Lundqvist C, Boriani G, Castella M, Dan GA, Dilaveris PE, Fauchier L, Filippatos G, Kalman JM, La Meir M, Lane DA, Lebeau JP, Lettino M, Lip GYH, Pinto FJ, Thomas GN, Valgimigli M, Van Gelder IC, Van Putte BP, Watkins CL; ESC Scientific Document Group. 2020 ESC Guidelines for the diagnosis and management of atrial fibrillation developed in collaboration with the European Association for Cardio-Thoracic Surgery (EACTS). Eur Heart J 2021;42:373–498. 32860505 10.1093/eurheartj/ehaa612

[6] Lip GY , Nieuwlaat R, Pisters R, Lane DA, Crijns HJ. Refining clinical risk stratification for predicting stroke and thromboembolism in atrial fibrillation using a novel risk factor-based approach: the euro heart survey on atrial fibrillation. Chest 2010;137:263–272. 19762550 10.1378/chest.09-1584

[7] Proietti M , Lane DA, Boriani G, Lip GYH. Stroke prevention, evaluation of bleeding risk, and anticoagulant treatment management in atrial fibrillation contemporary international guidelines. Can J Cardiol 2019;35:619–633. 31030864 10.1016/j.cjca.2019.02.009

[8] Rivera-Caravaca JM , Marín F, Vilchez JA, Gálvez J, Esteve-Pastor MA, Vicente V, Lip GYH, Roldán V. Refining stroke and bleeding prediction in atrial fibrillation by adding consecutive biomarkers to clinical risk scores. Stroke 2019;50:1372–1379. 31084333 10.1161/STROKEAHA.118.024305

[9] Boriani G , Diemberger I, Ziacchi M, Valzania C, Gardini B, Cimaglia P, Martignani C, Biffi M. AF burden is important—fact or fiction? Int J Clin Pract 2014;68:444–452. 24499075 10.1111/ijcp.12326

[10] Boriani G , Pettorelli D. Atrial fibrillation burden and atrial fibrillation type: clinical significance and impact on the risk of stroke and decision making for long-term anticoagulation. Vascul Pharmacol 2016;83:26–35. 27196706 10.1016/j.vph.2016.03.006

[11] Boriani G , Imberti JF, Vitolo M. Atrial fibrillation and remote monitoring through cardiac implantable electronic devices in heart failure patients. Eur J Heart Fail 2020;22:554–556. 31977147 10.1002/ejhf.1745

[12] Boriani G , Vitolo M. Atrial fibrillation in patients with cardiac implantable electronic devices: new perspectives with important clinical implications. Kardiol Pol 2019;77:1119–1120. 31855194 10.33963/KP.15110

[13] Freedman B , Camm J, Calkins H, Healey JS, Rosenqvist M, Wang J, Albert CM, Anderson CS, Antoniou S, Benjamin EJ, Boriani G, Brachmann J, Brandes A, Chao TF, Conen D, Engdahl J, Fauchier L, Fitzmaurice DA, Friberg L, Gersh BJ, Gladstone DJ, Glotzer TV, Gwynne K, Hankey GJ, Harbison J, Hillis GS, Hills MT, Kamel H, Kirchhof P, Kowey PR, Krieger D, Lee VWY, Levin L, Lip GYH, Lobban T, Lowres N, Mairesse GH, Martinez C, Neubeck L, Orchard J, Piccini JP, Poppe K, Potpara TS, Puererfellner H, Rienstra M, Sandhu RK, Schnabel RB, Siu CW, Steinhubl S, Svendsen JH, Svennberg E, Themistoclakis S, Tieleman RG, Turakhia MP, Tveit A, Uittenbogaart SB, Van Gelder IC, Verma A, Wachter R, Yan BP; AF-Screen Collaborators. Screening for atrial fibrillation: a report of the AF-SCREEN International Collaboration. Circulation 2017;135:1851–1867. 28483832 10.1161/CIRCULATIONAHA.116.026693

[14] Potpara TS , Lip GYH, Blomstrom-Lundqvist C, Boriani G, Van Gelder IC, Heidbuchel H, Hindricks G, Camm AJ. The 4S-AF scheme (Stroke Risk; Symptoms; Severity of Burden; Substrate): a novel approach to in-depth characterization (rather than classification) of atrial fibrillation. Thromb Haemost 2021;121:270–278. 32838473 10.1055/s-0040-1716408

[15] Chen LY , Chung MK, Allen LA, Ezekowitz M, Furie KL, McCabe P, Noseworthy PA, Perez MV, Turakhia MP; American Heart Association Council on Clinical Cardiology; Council on Cardiovascular and Stroke Nursing; Council on Quality of Care and Outcomes Research; and Stroke Council. Atrial fibrillation burden: moving beyond atrial fibrillation as a binary entity: a scientific statement from the American Heart Association. Circulation 2018;137:e623–e644. 29661944 10.1161/CIR.0000000000000568 PMC8463258

[16] Boriani G , Laroche C, Diemberger I, Fantecchi E, Popescu MI, Rasmussen LH, Dan GA, Kalarus Z, Tavazzi L, Maggioni AP, Lip GY. ‘Real-world’ management and outcomes of patients with paroxysmal vs. non-paroxysmal atrial fibrillation in Europe: the EURObservational Research Programme-Atrial Fibrillation (EORP-AF) General Pilot Registry. Europace 2016;18:648–657. 26826133 10.1093/europace/euv390

[17] Ganesan AN , Chew DP, Hartshorne T, Selvanayagam JB, Aylward PE, Sanders P, McGavigan AD. The impact of atrial fibrillation type on the risk of thromboembolism, mortality, and bleeding: a systematic review and meta-analysis. Eur Heart J 2016;37:1591–1602. 26888184 10.1093/eurheartj/ehw007

[18] Vanassche T , Lauw MN, Eikelboom JW, Healey JS, Hart RG, Alings M, Avezum A, Diaz R, Hohnloser SH, Lewis BS, Shestakovska O, Wang J, Connolly SJ. Risk of ischaemic stroke according to pattern of atrial fibrillation: analysis of 6563 aspirin-treated patients in ACTIVE-A and AVERROES. Eur Heart J 2015;36:281–287a. 25187524 10.1093/eurheartj/ehu307

[19] Diederichsen SZ , Haugan KJ, Brandes A, Lanng MB, Graff C, Krieger D, Kronborg C, Holst AG, Køber L, Højberg S, Svendsen JH. Natural history of subclinical atrial fibrillation detected by implanted loop recorders. J Am Coll Cardiol 2019;74:2771–2781. 31779791 10.1016/j.jacc.2019.09.050

[20] Boriani G , Glotzer TV, Santini M, West TM, De Melis M, Sepsi M, Gasparini M, Lewalter T, Camm JA, Singer DE. Device-detected atrial fibrillation and risk for stroke: an analysis of >10,000 patients from the SOS AF project (Stroke preventiOn Strategies based on Atrial Fibrillation information from implanted devices). Eur Heart J 2014;35:508–516. 24334432 10.1093/eurheartj/eht491 PMC3930873

[21] Freedman B , Boriani G, Glotzer TV, Healey JS, Kirchhof P, Potpara TS. Management of atrial high-rate episodes detected by cardiac implanted electronic devices. Nat Rev Cardiol 2017;14:701–714. 28682320 10.1038/nrcardio.2017.94

[22] Mahajan R , Perera T, Elliott AD, Twomey DJ, Kumar S, Munwar DA, Khokhar KB, Thiyagarajah A, Middeldorp ME, Nalliah CJ, Hendriks JML, Kalman JM, Lau DH, Sanders P. Subclinical device-detected atrial fibrillation and stroke risk: a systematic review and meta-analysis. Eur Heart J 2018;39:1407–1415. 29340587 10.1093/eurheartj/ehx731

[23] Steinberg BA , Piccini JP. When low-risk atrial fibrillation is not so low risk: beast of burden. JAMA Cardiol 2018;3:558–560. 29799958 10.1001/jamacardio.2018.1205

[24] Boriani G , Healey JS, Schnabel RB, Lopes RD, Calkins H, Camm JA, Freedman B. Oral anticoagulation for subclinical atrial tachyarrhythmias detected by implantable cardiac devices: an international survey of the AF-SCREEN Group. Int J Cardiol 2019;296:65–70. 31327519 10.1016/j.ijcard.2019.07.039

[25] Perino AC , Fan J, Askari M, Heidenreich PA, Keung E, Raitt MH, Piccini JP, Ziegler PD, Turakhia MP. Practice variation in anticoagulation prescription and outcomes after device-detected atrial fibrillation. Circulation 2019;139:2502–2512. 30880434 10.1161/CIRCULATIONAHA.118.038988 PMC6652191

[26] Lopes RD , Alings M, Connolly SJ, Beresh H, Granger CB, Mazuecos JB, Boriani G, Nielsen JC, Conen D, Hohnloser SH, Mairesse GH, Mabo P, Camm AJ, Healey JS. Rationale and design of the Apixaban for the Reduction of Thrombo-Embolism in Patients With Device-Detected Sub-Clinical Atrial Fibrillation (ARTESiA) trial. Am Heart J 2017;189:137–145. 28625370 10.1016/j.ahj.2017.04.008

[27] Kirchhof P , Blank BF, Calvert M, Camm AJ, Chlouverakis G, Diener HC, Goette A, Huening A, Lip GYH, Simantirakis E, Vardas P. Probing oral anticoagulation in patients with atrial high rate episodes: rationale and design of the Non-vitamin K antagonist Oral anticoagulants in patients with Atrial High rate episodes (NOAH-AFNET 6) trial. Am Heart J 2017;190:12–18. 28760205 10.1016/j.ahj.2017.04.015 PMC5546174

[28] Boriani G , Vitolo M, Imberti JF, Potpara TS, Lip GYH. What do we do about atrial high rate episodes? Eur Heart J Suppl 2020;22:O42–O52. 33380943 10.1093/eurheartj/suaa179 PMC7753882

[29] Boriani G , Glotzer TV, Ziegler PD, De Melis M, Mangoni di S Stefano L, Sepsi M, Landolina M, Lunati M, Lewalter T, Camm AJ. Detection of new atrial fibrillation in patients with cardiac implanted electronic devices and factors associated with transition to higher device-detected atrial fibrillation burden. Heart Rhythm 2018;15:376–383. 29122724 10.1016/j.hrthm.2017.11.007

[30] De With RR , Erküner Ö, Rienstra M, Nguyen BO, Körver FWJ, Linz D, Cate Ten H, Spronk H, Kroon AA, Maass AH, Blaauw Y, Tieleman RG, Hemels MEW, de Groot JR, Elvan A, de Melis M, Scheerder COS, Al-Jazairi MIH, Schotten U, Luermans JGLM, Crijns HJGM, Van Gelder IC; for the RACE V Investigators. Temporal patterns and short-term progression of paroxysmal atrial fibrillation: data from RACE V. Europace 2020;22:1162–1172. 32642768 10.1093/europace/euaa123 PMC7400474

[31] Larsen BS , Kumarathurai P, Falkenberg J, Nielsen OW, Sajadieh A. Excessive atrial ectopy and short atrial runs increase the risk of stroke beyond incident atrial fibrillation. J Am Coll Cardiol 2015;66:232–241. 26184616 10.1016/j.jacc.2015.05.018

[32] Charitos EI , Stierle U, Ziegler PD, Baldewig M, Robinson DR, Sievers HH, Hanke T. A comprehensive evaluation of rhythm monitoring strategies for the detection of atrial fibrillation recurrence: insights from 647 continuously monitored patients and implications for monitoring after therapeutic interventions. Circulation 2012;126:806–814. 22824434 10.1161/CIRCULATIONAHA.112.098079

[33] Al-Gibbawi M , Ayinde HO, Bhatia NK, El-Chami MF, Westerman SB, Leon AR, Shah AD, Patel AM, De Lurgio DB, Tompkins CM, Lloyd MS, Merchant FM, Kiani S. Relationship between device-detected burden and duration of atrial fibrillation and risk of ischemic stroke. Heart Rhythm 2021;18:338–346. 33250442 10.1016/j.hrthm.2020.10.017

[34] Boriani G , Botto GL, Padeletti L, Santini M, Capucci A, Gulizia M, Ricci R, Biffi M, De Santo T, Corbucci G, Lip GY; for the Italian AT-500 Registry Investigators. Improving stroke risk stratification using the CHADS_2_ and CHA_2_DS_2_-VASc risk scores in patients with paroxysmal atrial fibrillation by continuous arrhythmia burden monitoring. Stroke 2011;42:1768–1770. 21493904 10.1161/STROKEAHA.110.609297

[35] Kaplan RM , Koehler J, Ziegler PD, Sarkar S, Zweibel S, Passman RS. Stroke risk as a function of atrial fibrillation duration and CHA_2_DS_2_-VASc score. Circulation 2019;140:1639–1646. 31564126 10.1161/CIRCULATIONAHA.119.041303

[36] Diederichsen SZ , Haugan KJ, Brandes A, Graff C, Krieger D, Kronborg C, Holst AG, Nielsen JB, Køber L, Højberg S, Svendsen JH. Incidence and predictors of atrial fibrillation episodes as detected by implantable loop recorder in patients at risk: from the LOOP study. Am Heart J 2020;219:117–127. 31699295 10.1016/j.ahj.2019.09.009

[37] Nasir JM , Pomeroy W, Marler A, Hann M, Baykaner T, Jones R, Stoll R, Hursey K, Meadows A, Walker J, Kindsvater S. Predicting determinants of atrial fibrillation or flutter for therapy elucidation in patients at risk for thromboembolic events (PREDATE AF) study. Heart Rhythm 2017;14:955–961. 28506913 10.1016/j.hrthm.2017.04.026

[38] Reiffel JA , Verma A, Kowey PR, Halperin JL, Gersh BJ, Wachter R, Pouliot E, Ziegler PD; REVEAL AF Investigators. Incidence of previously undiagnosed atrial fibrillation using insertable cardiac monitors in a high-risk population: the REVEAL AF study. JAMA Cardiol 2017;2:1120–1127. 28842973 10.1001/jamacardio.2017.3180 PMC5710506

[39] Tarakji KG , Silva J, Chen LY, Turakhia MP, Perez M, Attia ZI, Passman R, Boissy A, Cho DJ, Majmudar M, Mehta N, Wan EY, Chung M. Digital health and the care of the patient with arrhythmia: what every electrophysiologist needs to know. Circ Arrhythm Electrophysiol 2020;13:e007953. 33021815 10.1161/CIRCEP.120.007953

[40] Boriani G , Schnabel RB, Healey JS, Lopes RD, Verbiest-van Gurp N, Lobban T, Camm JA, Freedman B. Consumer-led screening for atrial fibrillation using consumer-facing wearables, devices and apps: a survey of health care professionals by AF-SCREEN international collaboration. Eur J Intern Med 2020;82:97–104. 32933842 10.1016/j.ejim.2020.09.005

[41] Turakhia MP , Ziegler PD, Schmitt SK, Chang Y, Fan J, Than CT, Keung EK, Singer DE. Atrial fibrillation burden and short-term risk of stroke: case-crossover analysis of continuously recorded heart rhythm from cardiac electronic implanted devices. Circ Arrhythm Electrophysiol 2015;8:1040–1047. 26175528 10.1161/CIRCEP.114.003057

[42] Kamel H , Okin PM, Elkind MS, Iadecola C. Atrial fibrillation and mechanisms of stroke: time for a new model. Stroke 2016;47:895–900. 26786114 10.1161/STROKEAHA.115.012004 PMC4766055

[43] Brambatti M , Connolly SJ, Gold MR, Morillo CA, Capucci A, Muto C, Lau CP, Van Gelder IC, Hohnloser SH, Carlson M, Fain E, Nakamya J, Mairesse GH, Halytska M, Deng WQ, Israel CW, Healey JS; ASSERT Investigators. Temporal relationship between subclinical atrial fibrillation and embolic events. Circulation 2014;129:2094–2099. 24633881 10.1161/CIRCULATIONAHA.113.007825

[44] Goette A , Kalman JM, Aguinaga L, Akar J, Cabrera JA, Chen SA, Chugh SS, Corradi D, D'Avila A, Dobrev D, Fenelon G, Gonzalez M, Hatem SN, Helm R, Hindricks G, Ho SY, Hoit B, Jalife J, Kim YH, Lip GY, Ma CS, Marcus GM, Murray K, Nogami A, Sanders P, Uribe W, Van Wagoner DR, Nattel S; Document Reviewers. EHRA/HRS/APHRS/SOLAECE expert consensus on atrial cardiomyopathies: definition, characterization, and clinical implication. Europace 2016;18:1455–1490. 27402624 10.1093/europace/euw161 PMC6392440

[45] Bisbal F , Baranchuk A, Braunwald E, Bayés de Luna A, Bayés-Genís A. Atrial failure as a clinical entity: JACC review topic of the week. J Am Coll Cardiol 2020;75:222–232. 31948652 10.1016/j.jacc.2019.11.013

[46] Vaziri SM , Larson MG, Benjamin EJ, Levy D. Echocardiographic predictors of nonrheumatic atrial fibrillation. The Framingham Heart Study. Circulation 1994;89:724–730. 8313561 10.1161/01.cir.89.2.724

[47] Psaty BM , Manolio TA, Kuller LH, Kronmal RA, Cushman M, Fried LP, White R, Furberg CD, Rautaharju PM. Incidence of and risk factors for atrial fibrillation in older adults. Circulation 1997;96:2455–2461. 9337224 10.1161/01.cir.96.7.2455

[48] Tsang TS , Barnes ME, Bailey KR, Leibson CL, Montgomery SC, Takemoto Y, Diamond PM, Marra MA, Gersh BJ, Wiebers DO, Petty GW, Seward JB. Left atrial volume: important risk marker of incident atrial fibrillation in 1655 older men and women. Mayo Clinic Proc 2001;76:467–475. 10.4065/76.5.467 11357793

[49] Tsang TS , Abhayaratna WP, Barnes ME, Miyasaka Y, Gersh BJ, Bailey KR, Cha SS, Seward JB. Prediction of cardiovascular outcomes with left atrial size: is volume superior to area or diameter? J Am Coll Cardiol 2006;47:1018–1023. 16516087 10.1016/j.jacc.2005.08.077

[50] Abhayaratna WP , Fatema K, Barnes ME, Seward JB, Gersh BJ, Bailey KR, Casaclang-Verzosa G, Tsang TS. Left atrial reservoir function as a potent marker for first atrial fibrillation or flutter in persons > or = 65 years of age. Am J Cardiol 2008;101:1626–1629. 18489941 10.1016/j.amjcard.2008.01.051

[51] Patel RB , Delaney JA, Hu M, Patel H, Cheng J, Gottdiener J, Kizer JR, Marcus GM, Turakhia MP, Deo R, Heckbert SR, Psaty BM, Shah SJ. Characterization of cardiac mechanics and incident atrial fibrillation in participants of the Cardiovascular Health Study. JCI Insight 2020;5:10.1172/jci.insight.141656 PMC7566702 32910807

[52] Allessie M , Ausma J, Schotten U. Electrical, contractile and structural remodeling during atrial fibrillation. Cardiovasc Res 2002;54:230–246. 12062329 10.1016/s0008-6363(02)00258-4

[53] De With RR , Marcos EG, Van Gelder IC, Rienstra M. Atrial fibrillation progression and outcome in patients with young-onset atrial fibrillation. Europace 2018;20:1750–1757. 29518195 10.1093/europace/euy028

[54] Malavasi VL , Fantecchi E, Tordoni V, Melara L, Barbieri A, Vitolo M, Lip GYH, Boriani G. Atrial fibrillation pattern and factors affecting the progression to permanent atrial fibrillation. Intern Emerg Med 2020. 10.1007/s11739-020-02551-5 33161524

[55] Benjamin EJ , D'Agostino RB, Belanger AJ, Wolf PA, Levy D. Left atrial size and the risk of stroke and death. The Framingham Heart Study. Circulation 1995;92:835–841. 7641364 10.1161/01.cir.92.4.835

[56] Yaghi S , Moon YP, Mora-McLaughlin C, Willey JZ, Cheung K, Di Tullio MR, Homma S, Kamel H, Sacco RL, Elkind MS. Left atrial enlargement and stroke recurrence: the Northern Manhattan Stroke Study. Stroke 2015;46:1488–1493. 25908460 10.1161/STROKEAHA.115.008711 PMC4442058

[57] Inciardi RM , Rossi A. Left atrium: a forgotten biomarker and a potential target in cardiovascular medicine. J Cardiovasc Med (Hagerstown) 2019;20:797–808. 31609849 10.2459/JCM.0000000000000886

[58] Kamel H , Bartz TM, Elkind MSV, Okin PM, Thacker EL, Patton KK, Stein PK, deFilippi CR, Gottesman RF, Heckbert SR, Kronmal RA, Soliman EZ, Longstreth WT. Atrial cardiopathy and the risk of ischemic stroke in the CHS (Cardiovascular Health Study). Stroke 2018;49:980–986. 29535268 10.1161/STROKEAHA.117.020059 PMC5973804

[59] Affan M , Mahajan A, Modi S, Schultz L, Katramados A, Mayer SA, Miller DJ. Atrial fibrillation, not atrial cardiopathy, is associated with stroke: a single center retrospective study. J Neurolog Sci 2019;402:69–73. 10.1016/j.jns.2019.05.012 31121533

[60] Schotten U , Duytschaever M, Ausma J, Eijsbouts S, Neuberger HR, Allessie M. Electrical and contractile remodeling during the first days of atrial fibrillation go hand in hand. Circulation 2003;107:1433–1439. 12642366 10.1161/01.cir.0000055314.10801.4f

[61] Khan IA. Transient atrial mechanical dysfunction (stunning) after cardioversion of atrial fibrillation and flutter. Am Heart J 2002;144:11–22. 12094183 10.1067/mhj.2002.123113

[62] Manning WJ , Silverman DI, Katz SE, Riley MF, Come PC, Doherty RM, Munson JT, Douglas PS. Impaired left atrial mechanical function after cardioversion: relation to the duration of atrial fibrillation. J Am Coll Cardiol 1994;23:1535–1540. 8195510 10.1016/0735-1097(94)90652-1

[63] Kottkamp H , Schreiber D. The substrate in "early persistent" atrial fibrillation: arrhythmia induced, risk factor induced, or from a specific fibrotic atrial cardiomyopathy? JACC Clin Electrophysiol 2016;2:140–142. 29766862 10.1016/j.jacep.2016.02.010

[64] Nattel S. Molecular and cellular mechanisms of atrial fibrosis in atrial fibrillation. JACC Clin Electrophysiol 2017;3:425–435. 29759598 10.1016/j.jacep.2017.03.002

[65] Kornej J , Börschel CS, Benjamin EJ, Schnabel RB. Epidemiology of atrial fibrillation in the 21st century: novel methods and new insights. Circ Res 2020;127:4–20. 32716709 10.1161/CIRCRESAHA.120.316340 PMC7577553

[66] Andrade J , Khairy P, Dobrev D, Nattel S. The clinical profile and pathophysiology of atrial fibrillation: relationships among clinical features, epidemiology, and mechanisms. Circ Res 2014;114:1453–1468. 24763464 10.1161/CIRCRESAHA.114.303211

[67] Staerk L , Sherer JA, Ko D, Benjamin EJ, Helm RH. Atrial fibrillation: epidemiology, pathophysiology, and clinical outcomes. Circ Res 2017;120:1501–1517. 28450367 10.1161/CIRCRESAHA.117.309732 PMC5500874

[68] Benjamin EJ , Levy D, Vaziri SM, D'Agostino RB, Belanger AJ, Wolf PA. Independent risk factors for atrial fibrillation in a population-based cohort. The Framingham Heart Study. JAMA 1994;271:840–844. 8114238

[69] Farmakis D , Chrysohoou C, Giamouzis G, Giannakoulas G, Hamilos M, Naka K, Tzeis S, Xydonas S, Karavidas A, Parissis J. The management of atrial fibrillation in heart failure: an expert panel consensus. Heart Fail Rev 2020. 10.1007/s10741-020-09978-0 32468277

[70] Santhanakrishnan R , Wang N, Larson MG, Magnani JW, McManus DD, Lubitz SA, Ellinor PT, Cheng S, Vasan RS, Lee DS, Wang TJ, Levy D, Benjamin EJ, Ho JE. Atrial fibrillation begets heart failure and vice versa: temporal associations and differences in preserved versus reduced ejection fraction. Circulation 2016;133:484–492. 26746177 10.1161/CIRCULATIONAHA.115.018614 PMC4738087

[71] Komajda M , Anker SD, Cowie MR, Filippatos GS, Mengelle B, Ponikowski P, Tavazzi L; on behalf of the QUALIFY Investigators. Physicians' adherence to guideline-recommended medications in heart failure with reduced ejection fraction: data from the QUALIFY global survey. Eur J Heart Fail 2016;18:514–522. 27095461 10.1002/ejhf.510

[72] Ling LH , Kistler PM, Kalman JM, Schilling RJ, Hunter RJ. Comorbidity of atrial fibrillation and heart failure. Nat Rev Cardiol 2016;13:131–147. 26658575 10.1038/nrcardio.2015.191

[73] Blum S , Aeschbacher S, Meyre P, Zwimpfer L, Reichlin T, Beer JH, Ammann P, Auricchio A, Kobza R, Erne P, Moschovitis G, Di VM, Shah D, Schläpfer J, Henz S, Meyer-Zürn C, Roten L, Schwenkglenks M, Sticherling C, Kühne M, Osswald S, Conen D; Swiss‐AF Investigators. Incidence and predictors of atrial fibrillation progression. J Am Heart Assoc 2019;8:e012554. 31590581 10.1161/JAHA.119.012554 PMC6818023

[74] Carlisle MA , Fudim M, DeVore AD, Piccini JP. Heart failure and atrial fibrillation, like fire and fury. JACC Heart Fail 2019;7:447–456. 31146871 10.1016/j.jchf.2019.03.005

[75] Mogensen UM , Jhund PS, Abraham WT, Desai AS, Dickstein K, Packer M, Rouleau JL, Solomon SD, Swedberg K, Zile MR, Køber L, McMurray JJV; PARADIGM-HF and ATMOSPHERE Investigators and Committees. Type of atrial fibrillation and outcomes in patients with heart failure and reduced ejection fraction. J Am Coll Cardiol 2017;70:2490–2500. 29145948 10.1016/j.jacc.2017.09.027

[76] Wong JA , Conen D, Van Gelder IC, McIntyre WF, Crijns HJ, Wang J, Gold MR, Hohnloser SH, Lau CP, Capucci A, Botto G, Grönefeld G, Israel CW, Connolly SJ, Healey JS. Progression of device-detected subclinical atrial fibrillation and the risk of heart failure. J Am Coll Cardiol 2018;71:2603–2611. 29880119 10.1016/j.jacc.2018.03.519

[77] Wang TJ , Larson MG, Levy D, Vasan RS, Leip EP, Wolf PA, D'Agostino RB, Murabito JM, Kannel WB, Benjamin EJ. Temporal relations of atrial fibrillation and congestive heart failure and their joint influence on mortality: the Framingham Heart Study. Circulation 2003;107:2920–2925. 12771006 10.1161/01.CIR.0000072767.89944.6E

[78] Mamas MA , Caldwell JC, Chacko S, Garratt CJ, Fath-Ordoubadi F, Neyses L. A meta-analysis of the prognostic significance of atrial fibrillation in chronic heart failure. Eur J Heart Fail 2009;11:676–683. 19553398 10.1093/eurjhf/hfp085

[79] Siller-Matula JM , Pecen L, Patti G, Lucerna M, Kirchhof P, Lesiak M, Huber K, Verheugt FWA, Lang IM, Renda G, Schnabel RB, Wachter R, Kotecha D, Sellal JM, Rohla M, Ricci F, De Caterina R, TiA G. Heart failure subtypes and thromboembolic risk in patients with atrial fibrillation: the PREFER in AF-HF substudy. Int J Cardiol 2018;265:141–147. 29706429 10.1016/j.ijcard.2018.04.093

[80] Mentias A , Briasoulis A, Shantha G, Alvarez P, Vaughan-Sarrazin M. Impact of heart failure type on thromboembolic and bleeding risk in patients with atrial fibrillation on oral anticoagulation. Am J Cardiol 2019;123:1649–1653. 30928033 10.1016/j.amjcard.2019.02.027 PMC8263234

[81] Li D , Fareh S, Leung TK, Nattel S. Promotion of atrial fibrillation by heart failure in dogs: atrial remodeling of a different sort. Circulation 1999;100:87–95. 10393686 10.1161/01.cir.100.1.87

[82] Crijns HJ , Van den Berg MP, Van Gelder IC, Van Veldhuisen DJ. Management of atrial fibrillation in the setting of heart failure. Eur Heart J 1997;18(Suppl. C):C45–49. 9152675 10.1093/eurheartj/18.suppl_c.45

[83] Kirchhof P , Camm AJ, Goette A, Brandes A, Eckardt L, Elvan A, Fetsch T, van Gelder IC, Haase D, Haegeli LM, Hamann F, Heidbüchel H, Hindricks G, Kautzner J, Kuck KH, Mont L, Ng GA, Rekosz J, Schoen N, Schotten U, Suling A, Taggeselle J, Themistoclakis S, Vettorazzi E, Vardas P, Wegscheider K, Willems S, Crijns H, Breithardt G. Early rhythm-control therapy in patients with atrial fibrillation. N Engl J Med 2020;383:1305–1316. 32865375 10.1056/NEJMoa2019422

[84] Williams B , Mancia G, Spiering W, Agabiti Rosei E, Azizi M, Burnier M, Clement DL, Coca A, de Simone G, Dominiczak A, Kahan T, Mahfoud F, Redon J, Ruilope L, Zanchetti A, Kerins M, Kjeldsen SE, Kreutz R, Laurent S, Lip GYH, McManus R, Narkiewicz K, Ruschitzka F, Schmieder RE, Shlyakhto E, Tsioufis C, Aboyans V, Desormais I; ESC Scientific Document Group. 2018 ESC/ESH Guidelines for the management of arterial hypertension. Eur Heart J 2018;39:3021–3104. 30165516 10.1093/eurheartj/ehy339

[85] Kannel WB , Abbott RD, Savage DD, McNamara PM. Epidemiologic features of chronic atrial fibrillation: the Framingham study. N Engl J Med 1982;306:1018–1022. 7062992 10.1056/NEJM198204293061703

[86] Krahn AD , Manfreda J, Tate RB, Mathewson FA, Cuddy TE. The natural history of atrial fibrillation: incidence, risk factors, and prognosis in the Manitoba Follow-Up Study. Am J Med 1995;98:476–484. 7733127 10.1016/S0002-9343(99)80348-9

[87] Huxley RR , Lopez FL, Folsom AR, Agarwal SK, Loehr LR, Soliman EZ, Maclehose R, Konety S, Alonso A. Absolute and attributable risks of atrial fibrillation in relation to optimal and borderline risk factors: the Atherosclerosis Risk in Communities (ARIC) study. Circulation 2011;123:1501–1508. 21444879 10.1161/CIRCULATIONAHA.110.009035 PMC3181498

[88] Park YJ , Yang PS, Yu HT, Kim TH, Jang E, Uhm JS, Pak HN, Lee MH, Lip GYH, Joung B. What is the ideal blood pressure threshold for the prevention of atrial fibrillation in elderly general population? J Clin Med 2020;9: 2988. 10.3390/jcm9092988 PMC7563734 32947828

[89] Verdecchia P , Reboldi G, Gattobigio R, Bentivoglio M, Borgioni C, Angeli F, Carluccio E, Sardone MG, Porcellati C. Atrial fibrillation in hypertension: predictors and outcome. Hypertension 2003;41:218–223. 12574085 10.1161/01.hyp.0000052830.02773.e4

[90] Seko Y , Kato T, Haruna T, Izumi T, Miyamoto S, Nakane E, Inoko M. Association between atrial fibrillation, atrial enlargement, and left ventricular geometric remodeling. Sci Rep 2018;8:6366. 29686287 10.1038/s41598-018-24875-1 PMC5913256

[91] Chrispin J , Jain A, Soliman EZ, Guallar E, Alonso A, Heckbert SR, Bluemke DA, Lima JA, Nazarian S. Association of electrocardiographic and imaging surrogates of left ventricular hypertrophy with incident atrial fibrillation: MESA (Multi-Ethnic Study of Atherosclerosis). J Am Coll Cardiol 2014;63:2007–2013. 24657688 10.1016/j.jacc.2014.01.066 PMC4024364

[92] Wachtell K , Lehto M, Gerdts E, Olsen MH, Hornestam B, Dahlof B, Ibsen H, Julius S, Kjeldsen SE, Lindholm LH, Nieminen MS, Devereux RB. Angiotensin II receptor blockade reduces new-onset atrial fibrillation and subsequent stroke compared to atenolol: the Losartan Intervention For End Point Reduction in Hypertension (LIFE) study. J Am Coll Cardiol 2005;45:712–719. 15734615 10.1016/j.jacc.2004.10.068

[93] Okin PM , Wachtell K, Devereux RB, Harris KE, Jern S, Kjeldsen SE, Julius S, Lindholm LH, Nieminen MS, Edelman JM, Hille DA, Dahlof B. Regression of electrocardiographic left ventricular hypertrophy and decreased incidence of new-onset atrial fibrillation in patients with hypertension. JAMA 2006;296:1242–1248. 16968848 10.1001/jama.296.10.1242

[94] De Vos CB , Breithardt G, Camm AJ, Dorian P, Kowey PR, Le Heuzey JY, Naditch-Brule L, Prystowsky EN, Schwartz PJ, Torp-Pedersen C, Weintraub WS, Crijns HJ. Progression of atrial fibrillation in the REgistry on Cardiac rhythm disORDers assessing the control of Atrial Fibrillation cohort: clinical correlates and the effect of rhythm-control therapy. Am Heart J 2012;163:887–893. 22607868 10.1016/j.ahj.2012.02.015

[95] Erküner Ö , Dudink EAMP, Nieuwlaat R, Rienstra M, Van Gelder IC, Camm AJ, Capucci A, Breithardt G, LeHeuzey JY, Lip GYH, Crijns HJGM, Luermans JGLM. Effect of systemic hypertension with versus without left ventricular hypertrophy on the progression of atrial fibrillation (from the Euro Heart Survey). Am J Cardiol 2018;122:578–583. 29958714 10.1016/j.amjcard.2018.04.053

[96] Rider OJ , Lewandowski A, Nethononda R, Petersen SE, Francis JM, Pitcher A, Holloway CJ, Dass S, Banerjee R, Byrne JP, Leeson P, Neubauer S. Gender-specific differences in left ventricular remodelling in obesity: insights from cardiovascular magnetic resonance imaging. Eur Heart J 2013;34:292–299. 23053174 10.1093/eurheartj/ehs341 PMC3549525

[97] Lip GY , Frison L, Grind M; on behalf of the SPORTIF Investigators. Effect of hypertension on anticoagulated patients with atrial fibrillation. Eur Heart J 2007;28:752–759. 17289744 10.1093/eurheartj/ehl504

[98] Rao MP , Halvorsen S, Wojdyla D, Thomas L, Alexander JH, Hylek EM, Hanna M, Bahit MC, Lopes RD, De CR, Erol C, Goto S, Lanas F, Lewis BS, Husted S, Gersh BJ, Wallentin L, Granger CB; Apixaban for Reduction in Stroke and Other Thromboembolic Events in Atrial Fibrillation (ARISTOTLE) Steering Committee and Investigators. Blood pressure control and risk of stroke or systemic embolism in patients with atrial fibrillation: results from the Apixaban for Reduction in Stroke and Other Thromboembolic Events in Atrial Fibrillation (ARISTOTLE) trial. J Am Heart Assoc 2015;4:e002015. 26627878 10.1161/JAHA.115.002015 PMC4845276

[99] Vemulapalli S , Hellkamp AS, Jones WS, Piccini JP, Mahaffey KW, Becker RC, Hankey GJ, Berkowitz SD, Nessel CC, Breithardt G, Singer DE, Fox KA, Patel MR. Blood pressure control and stroke or bleeding risk in anticoagulated patients with atrial fibrillation: results from the ROCKET AF Trial. Am Heart J 2016;178:74–84. 27502854 10.1016/j.ahj.2016.05.001

[100] Aeschbacher S , Blum S, Meyre PB, Coslovsky M, Vischer AS, Sinnecker T, Rodondi N, Beer JH, Moschovitis G, Moutzouri E, Hunkeler C, Burkard T, Eken C, Roten L, Zuern CS, Sticherling C, Wuerfel J, Bonati LH, Conen D, Osswald S, Kühne M, Auberson C, Ceylan S, Doerpfeld S, Girod M, Hennings E, Krisai P, Monsch AU, Müller C, Springer A, Voellmin G, Aujesky D, Fischer U, Fuhrer J, Jung S, Mattle H, Adam L, Elodie Aubert C, Feller M, Loewe A, Schneider C, Flückiger T, Groen C, Ehrsam L, Hellrigl S, Nuoffer A, Rakovic D, Schwab N, Wenger R, Müller A, Beynon C, Dillier R, Deubelbeiss M, Eberli F, Franzini C, Juchli I, Liedtke C, Nadler J, Obst T, Roth J, Schlomowitsch F, Schneider X, Studerus K, Tynan N, Weishaupt D, Fontana S, Kuest S, Scheuch K, Hischier D, Bonetti N, Grau A, Villinger J, Laube E, Baumgartner P, Filipovic M, Frick M, Montrasio G, Leuenberger S, Rutz F, Moccetti T, Auricchio A, Anesini A, Camporini C, Conte G, Luce Caputo M, Regoli F, Ammann P, Brenner R, Altmann D, Gemperle M, Hayoz D, Firmann M, Foucras S, Rime M, Kobza R, Berte B, Justi V, Kellner-Weldon F, Mehmann B, Meier S, Roth M, Ruckli-Kaeppeli A, Russi I, Schmidt K, Young M, Zbinden M, Frangi-Kultalahti J, Pin A, Shah D, Ehret G, Gallet H, Guillermet E, Lazeyras F, Lovblad K-O, Perret P, Tavel P, Teres C, Schläpfer J, Lauriers N, Méan M, Salzmann S, Stephan F-P, Grêt A, Novak J, Vitelli S, Di Valentino M, Frangi-Kultalahti J, Gallino A, Witassek F, Schwenkglenks M, Altermatt A, Amann M, Huber P, Ruberte E, Zuber V, Benkert P, Dutilh G, Markovic M, Neuschwander P, Simon P, Schmid R. Blood pressure and brain lesions in patients with atrial fibrillation. Hypertension 2021;77:662–671. 33356398 10.1161/HYPERTENSIONAHA.120.16025 PMC7803457

[101] Vitolo M , Lip GYH, Shantsila A. Why is atrial fibrillation so frequent in hypertensive patients? Am J Hypertens 2020;33:1067–1070. 32965491 10.1093/ajh/hpaa157

[102] Gumprecht J , Domek M, Lip GYH, Shantsila A. Invited review: hypertension and atrial fibrillation: epidemiology, pathophysiology, and implications for management. J Hum Hypertens 2019;33:824–836. 31690818 10.1038/s41371-019-0279-7

[103] Schotten U , Verheule S, Kirchhof P, Goette A. Pathophysiological mechanisms of atrial fibrillation: a translational appraisal. Physiol Rev 2011;91:265–325. 21248168 10.1152/physrev.00031.2009

[104] Proietti M , Boriani G. Obesity paradox in atrial fibrillation: implications for outcomes and relationship with oral anticoagulant drugs. Am J Cardiovasc Drugs 2020;20:125–137. 31583532 10.1007/s40256-019-00374-0

[105] Wanahita N , Messerli FH, Bangalore S, Gami AS, Somers VK, Steinberg JS. Atrial fibrillation and obesity—results of a meta-analysis. Am Heart J 2008;155:310–315. 18215602 10.1016/j.ahj.2007.10.004

[106] Aune D , Sen A, Schlesinger S, Norat T, Janszky I, Romundstad P, Tonstad S, Riboli E, Vatten LJ. Body mass index, abdominal fatness, fat mass and the risk of atrial fibrillation: a systematic review and dose-response meta-analysis of prospective studies. Eur J Epidemiol 2017;32:181–192. 28194602 10.1007/s10654-017-0232-4 PMC5380695

[107] Guglin M , Maradia K, Chen R, Curtis AB. Relation of obesity to recurrence rate and burden of atrial fibrillation. Am J Cardiol 2011;107:579–582. 21195377 10.1016/j.amjcard.2010.10.018

[108] Tsang TS , Barnes ME, Miyasaka Y, Cha SS, Bailey KR, Verzosa GC, Seward JB, Gersh BJ. Obesity as a risk factor for the progression of paroxysmal to permanent atrial fibrillation: a longitudinal cohort study of 21 years. Eur Heart J 2008;29:2227–2233. 18611964 10.1093/eurheartj/ehn324 PMC2733739

[109] Badheka AO , Rathod A, Kizilbash MA, Garg N, Mohamad T, Afonso L, Jacob S. Influence of obesity on outcomes in atrial fibrillation: yet another obesity paradox. Am J Med 2010;123:646–651. 20609687 10.1016/j.amjmed.2009.11.026

[110] Boriani G , Ruff CT, Kuder JF, Shi M, Lanz HJ, Rutman H, Mercuri MF, Antman EM, Braunwald E, Giugliano RP. Relationship between body mass index and outcomes in patients with atrial fibrillation treated with edoxaban or warfarin in the ENGAGE AF-TIMI 48 trial. Eur Heart J 2019;40:1541–1550. 30624719 10.1093/eurheartj/ehy861

[111] Elagizi A , Kachur S, Lavie CJ, Carbone S, Pandey A, Ortega FB, Milani RV. An overview and update on obesity and the obesity paradox in cardiovascular diseases. Prog Cardiovasc Dis 2018;61:142–150. 29981771 10.1016/j.pcad.2018.07.003

[112] Zhu W , Wan R, Liu F, Hu J, Huang L, Li J, Hong K. Relation of body mass index with adverse outcomes among patients with atrial fibrillation: a meta-analysis and systematic review. JAHA 2016;5:e004006. 27613773 10.1161/JAHA.116.004006 PMC5079045

[113] Proietti M , Guiducci E, Cheli P, Lip GY. Is there an obesity paradox for outcomes in atrial fibrillation? A systematic review and meta-analysis of non-vitamin K antagonist oral anticoagulant trials. Stroke 2017;48:857–866. 28265017 10.1161/STROKEAHA.116.015984

[114] Lavie CJ , Pandey A, Lau DH, Alpert MA, Sanders P. Obesity and atrial fibrillation prevalence, pathogenesis, and prognosis: effects of weight loss and exercise. J Am Coll Cardiol 2017;70:2022–2035. 29025560 10.1016/j.jacc.2017.09.002

[115] Al-Rawahi M , Proietti R, Thanassoulis G. Pericardial fat and atrial fibrillation: epidemiology, mechanisms and interventions. Int J Cardiol 2015;195:98–103. 26025867 10.1016/j.ijcard.2015.05.129

[116] Huxley RR , Alonso A, Lopez FL, Filion KB, Agarwal SK, Loehr LR, Soliman EZ, Pankow JS, Selvin E. Type 2 diabetes, glucose homeostasis and incident atrial fibrillation: the Atherosclerosis Risk in Communities study. Heart 2012;98:133–138. 21930722 10.1136/heartjnl-2011-300503 PMC3237721

[117] Huxley RR , Filion KB, Konety S, Alonso A. Meta-analysis of cohort and case-control studies of type 2 diabetes mellitus and risk of atrial fibrillation. Am J Cardiol 2011;108:56–62. 21529739 10.1016/j.amjcard.2011.03.004 PMC3181495

[118] Magnussen C , Niiranen TJ, Ojeda FM, Gianfagna F, Blankenberg S, Njølstad I, Vartiainen E, Sans S, Pasterkamp G, Hughes M, Costanzo S, Donati MB, Jousilahti P, Linneberg A, Palosaari T, de Gaetano G, Bobak M, den Ruijter HM, Mathiesen E, Jørgensen T, Söderberg S, Kuulasmaa K, Zeller T, Iacoviello L, Salomaa V, Schnabel RB; BiomarCaRE Consortium. Sex differences and similarities in atrial fibrillation epidemiology, risk factors, and mortality in community cohorts: results from the BiomarCaRE Consortium (Biomarker for Cardiovascular Risk Assessment in Europe). Circulation 2017;136:1588–1597. 29038167 10.1161/CIRCULATIONAHA.117.028981 PMC5657474

[119] Fumagalli S , Said SA, Laroche C, Gabbai D, Boni S, Marchionni N, Boriani G, Maggioni AP, Musialik-Lydka A, Sokal A, Petersen J, Crijns HJGM, Lip GYH; the EORP-AF General Pilot Registry Investigators. Management and prognosis of atrial fibrillation in diabetic patients: an EORP-AF General Pilot Registry report. Eur Heart J Cardiovasc Pharmacother 2018;4:172–179. 29309557 10.1093/ehjcvp/pvx037

[120] Echouffo-Tcheugui JB , Shrader P, Thomas L, Gersh BJ, Kowey PR, Mahaffey KW, Singer DE, Hylek EM, Go AS, Peterson ED, Piccini JP, Fonarow GC. Care patterns and outcomes in atrial fibrillation patients with and without diabetes: ORBIT-AF registry. J Am Coll Cardiol 2017;70:1325–1335. 28882229 10.1016/j.jacc.2017.07.755

[121] Wang A , Green JB, Halperin JL, Piccini JP. Atrial fibrillation and diabetes mellitus: JACC review topic of the week. J Am Coll Cardiol 2019;74:1107–1115. 31439220 10.1016/j.jacc.2019.07.020

[122] Grisanti LA. Diabetes and arrhythmias: pathophysiology, mechanisms and therapeutic outcomes. Front Physiol 2018;9:1669. 30534081 10.3389/fphys.2018.01669 PMC6275303

[123] Sato H , Hosojima M, Ishikawa T, Aoki K, Okamoto T, Saito A, Tsuchida M. Glucose variability based on continuous glucose monitoring assessment is associated with postoperative complications after cardiovascular surgery. Ann Thorac Cardiovasc Surg 2017;23:239–247. 28717057 10.5761/atcs.oa.17-00045 PMC5655336

[124] Boriani G , Savelieva I, Dan GA, Deharo JC, Ferro C, Israel CW, Lane DA, La Manna G, Morton J, Mitjans AM, Vos MA, Turakhia MP, Lip GY; Document reviewers. Chronic kidney disease in patients with cardiac rhythm disturbances or implantable electrical devices: clinical significance and implications for decision making—a position paper of the European Heart Rhythm Association endorsed by the Heart Rhythm Society and the Asia Pacific Heart Rhythm Society. Europace 2015;17:1169–1196. 26108808 10.1093/europace/euv202 PMC6281310

[125] Soliman EZ , Prineas RJ, Go AS, Xie D, Lash JP, Rahman M, Ojo A, Teal VL, Jensvold NG, Robinson NL, Dries DL, Bazzano L, Mohler ER, Wright JT, Feldman HI, Group C. Chronic kidney disease and prevalent atrial fibrillation: the Chronic Renal Insufficiency Cohort (CRIC). Am Heart J 2010;159:1102–1107. 20569726 10.1016/j.ahj.2010.03.027 PMC2891979

[126] Carrero JJ , Trevisan M, Sood MM, Bárány P, Xu H, Evans M, Friberg L, Szummer K. Incident atrial fibrillation and the risk of stroke in adults with chronic kidney disease: the Stockholm CREAtinine Measurements (SCREAM) Project. CJASN 2018;13:1314–1320. 30030271 10.2215/CJN.04060318 PMC6140568

[127] Watanabe H , Watanabe T, Sasaki S, Nagai K, Roden DM, Aizawa Y. Close bidirectional relationship between chronic kidney disease and atrial fibrillation: the Niigata preventive medicine study. Am Heart J 2009;158:629–636. 19781424 10.1016/j.ahj.2009.06.031

[128] Boriani G , Laroche C, Diemberger I, Popescu MI, Rasmussen LH, Petrescu L, Crijns HJGM, Tavazzi L, Maggioni AP, Lip GYH. Glomerular filtration rate in patients with atrial fibrillation and 1-year outcomes. Sci Rep 2016;6:30271. 27466080 10.1038/srep30271 PMC4964613

[129] Diemberger I , Genovesi S, Massaro G, Reggiani MLB, Frisoni J, Gorlato G, Mauro E, Padeletti M, Vincenti A, Boriani G. Meta-analysis of clinical outcomes of electrical cardioversion and catheter ablation in patients with atrial fibrillation and chronic kidney disease. CPD 2018;24:2794–2801. 10.2174/1381612824666180829112019 30156153

[130] Ding WY , Gupta D, Wong CF, Lip GYH. Pathophysiology of atrial fibrillation and chronic kidney disease. Cardiovasc Res 2020;117:1046–1059. 10.1093/cvr/cvaa258 32871005

[131] Providência R , Marijon E, Boveda S, Barra S, Narayanan K, Le Heuzey JY, Gersh BJ, Gonçalves L. Meta-analysis of the influence of chronic kidney disease on the risk of thromboembolism among patients with nonvalvular atrial fibrillation. Am J Cardiol 2014;114:646–653. 25001152 10.1016/j.amjcard.2014.05.048

[132] Banerjee A , Fauchier L, Vourc'h P, Andres CR, Taillandier S, Halimi JM, Lip GY. Renal impairment and ischemic stroke risk assessment in patients with atrial fibrillation: the Loire Valley Atrial Fibrillation Project. J Am Coll Cardiol 2013;61:2079–2087. 23524209 10.1016/j.jacc.2013.02.035

[133] Roldán V , Marín F, Manzano-Fernandez S, Fernández H, Gallego P, Valdés M, Vicente V, Lip GY. Does chronic kidney disease improve the predictive value of the CHADS2 and CHA2DS2-VASc stroke stratification risk scores for atrial fibrillation? Thromb Haemost 2013;109:956–960. 23572113 10.1160/TH13-01-0054

[134] Goette A , Staack T, Rocken C, Arndt M, Geller JC, Huth C, Ansorge S, Klein HU, Lendeckel U. Increased expression of extracellular signal-regulated kinase and angiotensin-converting enzyme in human atria during atrial fibrillation. J Am Coll Cardiol 2000;35:1669–1677. 10807475 10.1016/s0735-1097(00)00611-2

[135] Qiu H , Ji C, Liu W, Wu Y, Lu Z, Lin Q, Xue Z, Liu X, Wu H, Jiang W, Zou C. Chronic kidney disease increases atrial fibrillation inducibility: involvement of inflammation, atrial fibrosis, and connexins. Front Physiol 2018;9:1726. 30564139 10.3389/fphys.2018.01726 PMC6288485

[136] Fukunaga N , Takahashi N, Hagiwara S, Kume O, Fukui A, Teshima Y, Shinohara T, Nawata T, Hara M, Noguchi T, Saikawa T. Establishment of a model of atrial fibrillation associated with chronic kidney disease in rats and the role of oxidative stress. Heart Rhythm 2012;9:2023–2031. 22906534 10.1016/j.hrthm.2012.08.019

[137] Michniewicz E , Mlodawska E, Lopatowska P, Tomaszuk-Kazberuk A, Malyszko J. Patients with atrial fibrillation and coronary artery disease—double trouble. Adv Med Sci 2018;63:30–35. 28818746 10.1016/j.advms.2017.06.005

[138] Diemberger I , Fantecchi E, Reggiani MLB, Martignani C, Angeletti A, Massaro G, Ziacchi M, Biffi M, Lip GYH, Boriani G. Atrial fibrillation and prediction of mortality by conventional clinical score systems according to the setting of care. Int J Cardiol 2018;261:73–77. 29572083 10.1016/j.ijcard.2018.03.058

[139] Heeringa J , van der Kuip DA, Hofman A, Kors JA, van Rooij FJ, Lip GY, Witteman JC. Subclinical atherosclerosis and risk of atrial fibrillation: the Rotterdam study. Arch Intern Med 2007;167:382–387. 17325300 10.1001/archinte.167.4.382

[140] Vinter N , Christesen AMS, Mortensen LS, Urbonaviciene G, Lindholt J, Johnsen SP, Frost L. Coronary artery calcium score and the long-term risk of atrial fibrillation in patients undergoing non-contrast cardiac computed tomography for suspected coronary artery disease: a Danish registry-based cohort study. Eur Heart J Cardiovasc Imaging 2018;19:926–932. 28977363 10.1093/ehjci/jex201

[141] Vallabhajosyula S , Patlolla SH, Verghese D, Ya'Qoub L, Kumar V, Subramaniam AV, Cheungpasitporn W, Sundaragiri PR, Noseworthy PA, Mulpuru SK, Bell MR, Gersh BJ, Deshmukh AJ. Burden of arrhythmias in acute myocardial infarction complicated by cardiogenic shock. Am J Cardiol 2020;125:1774–1781. 32307093 10.1016/j.amjcard.2020.03.015 PMC7261623

[142] Eldar M , Canetti M, Rotstein Z, Boyko V, Gottlieb S, Kaplinsky E, Behar S. Significance of paroxysmal atrial fibrillation complicating acute myocardial infarction in the thrombolytic era. SPRINT and Thrombolytic Survey Groups. Circulation 1998;97:965–970. 9529264 10.1161/01.cir.97.10.965

[143] Crenshaw BS , Ward SR, Granger CB, Stebbins AL, Topol EJ, Califf RM. Atrial fibrillation in the setting of acute myocardial infarction: the GUSTO-I experience. Global Utilization of Streptokinase and TPA for Occluded Coronary Arteries. J Am Coll Cardiol 1997;30:406–413. 9247512 10.1016/s0735-1097(97)00194-0

[144] Mercado-Lubo R , Yarzebski J, Lessard D, Gore J, Goldberg RJ. Changing trends in the landscape of patients hospitalized with acute myocardial infarction (2001 to 2011) (from the Worcester Heart Attack Study). Am J Cardiol 2020;125:673–677. 31924320 10.1016/j.amjcard.2019.12.009 PMC7160648

[145] Siu CW , Jim MH, Ho HH, Miu R, Lee SW, Lau CP, Tse HF. Transient atrial fibrillation complicating acute inferior myocardial infarction: implications for future risk of ischemic stroke. Chest 2007;132:44–49. 17400657 10.1378/chest.06-2733

[146] Lip G , Freedman B, De Caterina R, Potpara TS. Stroke prevention in atrial fibrillation: past, present and future. Comparing the guidelines and practical decision-making. Thromb Haemost 2017;117:1230–1239. 28597905 10.1160/TH16-11-0876

[147] Steensig K , Olesen KKW, Thim T, Nielsen JC, Jensen SE, Jensen LO, Kristensen SD, Bøtker HE, Lip GYH, Maeng M. Should the presence or extent of coronary artery disease be quantified in the CHA2DS2-VASc score in atrial fibrillation? A report from the Western Denmark Heart Registry. Thromb Haemost 2018;118:2162–2170. 30419601 10.1055/s-0038-1675401

[148] Olesen KKW , Steensig K, Madsen M, Thim T, Jensen LO, Raungaard B, Eikelboom J, Kristensen SD, Botker HE, Maeng M. Comparison of frequency of ischemic stroke in patients with versus without coronary heart disease and without atrial fibrillation. Am J Cardiol 2019;123:153–158. 30389089 10.1016/j.amjcard.2018.09.011

[149] Verheugt FWA , Ambrosio G, Atar D, Bassand JP, Camm AJ, Costabel JP, Fitzmaurice DA, Illingworth L, Goldhaber SZ, Goto S, Haas S, Jansky P, Kayani G, Stepinska J, Turpie AGG, van Eickels M, Kakkar AK; GARFIELD-AF Investigators. Outcomes in newly diagnosed atrial fibrillation and history of acute coronary syndromes: insights from GARFIELD-AF. Am J Med 2019;132:1431–1440.e7. 31306621 10.1016/j.amjmed.2019.06.008

[150] Cushing EH , Feil HS, Stanton EJ, Wartman WB. INFARCTION OF THE CARDIAC AURICLES (ATRIA): CLINICAL, PATHOLOGICAL, AND EXPERIMENTAL STUDIES. Br Heart J 1942;4:17–34. 18609908 10.1136/hrt.4.1-2.17 PMC503480

[151] Rivard L , Sinno H, Shiroshita-Takeshita A, Schram G, Leung TK, Nattel S. The pharmacological response of ischemia-related atrial fibrillation in dogs: evidence for substrate-specific efficacy. Cardiovasc Res 2007;74:104–113. 17316585 10.1016/j.cardiores.2007.01.018

[152] Criqui MH , Aboyans V. Epidemiology of peripheral artery disease. Circ Res 2015;116:1509–1526. 25908725 10.1161/CIRCRESAHA.116.303849

[153] Aboyans V , Ricco JB, Bartelink MEL, Björck M, Brodmann M, Cohnert T, Collet JP, Czerny M, De Carlo M, Debus S, Espinola-Klein C, Kahan T, Kownator S, Mazzolai L, Naylor AR, Roffi M, Röther J, Sprynger M, Tendera M, Tepe G, Venermo M, Vlachopoulos C, Desormais I; ESC Scientific Document Group. 2017 ESC Guidelines on the Diagnosis and Treatment of Peripheral Arterial Diseases, in collaboration with the European Society for Vascular Surgery (ESVS): document covering atherosclerotic disease of extracranial carotid and vertebral, mesenteric, renal, upper and lower extremity arteriesEndorsed by: the European Stroke Organization (ESO)The Task Force for the Diagnosis and Treatment of Peripheral Arterial Diseases of the European Society of Cardiology (ESC) and of the European Society for Vascular Surgery (ESVS). Eur Heart J 2018;39:763–816. 28886620 10.1093/eurheartj/ehx095

[154] Proietti M , Farcomeni A. Association between peripheral artery disease and incident risk of atrial fibrillation: strong evidence coming from population-based cohort studies. J Am Heart Assoc 2018;7:e009126. 29666067 10.1161/JAHA.118.009126 PMC6015415

[155] Proietti M , Raparelli V, Laroche C, Dan GA, Janion M, Popescu R, Sinagra G, Vijgen J, Boriani G, Maggioni AP, Tavazzi L, Lip GYH; on behalf of the EORP-AF Gen Pilot Investigators. Adverse outcomes in patients with atrial fibrillation and peripheral arterial disease: a report from the EURObservational research programme pilot survey on atrial fibrillation. Europace 2017;19:1439–1448. 27940934 10.1093/europace/euw169

[156] Proietti M , Calvieri C, Malatino L, Signorelli S, Corazza GR, Perticone F, Vestri AR, Loffredo L, Davì G, Violi F, Basili S; ARAPACIS (Atrial Fibrillation Registry for Ankle-Brachial Index Prevalence Assessment-Collaborative Italian Study) STUDY Investigators. Relationship between carotid intima-media thickness and non valvular atrial fibrillation type. Atherosclerosis 2015;238:350–355. 25555267 10.1016/j.atherosclerosis.2014.12.022

[157] Jones WS , Hellkamp AS, Halperin J, Piccini JP, Breithardt G, Singer DE, Fox KA, Hankey GJ, Mahaffey KW, Califf RM, Patel MR. Efficacy and safety of rivaroxaban compared with warfarin in patients with peripheral artery disease and non-valvular atrial fibrillation: insights from ROCKET AF. Eur Heart J 2014;35:242–249. 24302273 10.1093/eurheartj/eht492

[158] Heeringa J , van der Kuip DA, Hofman A, Kors JA, van Herpen G, Stricker BH, Stijnen T, Lip GY, Witteman JC. Prevalence, incidence and lifetime risk of atrial fibrillation: the Rotterdam study. Eur Heart J 2006;27:949–953. 16527828 10.1093/eurheartj/ehi825

[159] Collen J , Lettieri C, Wickwire E, Holley A. Obstructive sleep apnea and cardiovascular disease, a story of confounders!. Sleep Breath 2020;24:1299–1313. 31919716 10.1007/s11325-019-01945-w

[160] Mehra R , Benjamin EJ, Shahar E, Gottlieb DJ, Nawabit R, Kirchner HL, Sahadevan J, Redline S, Study SHH. Association of nocturnal arrhythmias with sleep-disordered breathing: the Sleep Heart Health Study. Am J Respir Crit Care Med 2006;173:910–916. 16424443 10.1164/rccm.200509-1442OC PMC2662909

[161] Bitter T , Westerheide N, Prinz C, Hossain MS, Vogt J, Langer C, Horstkotte D, Oldenburg O. Cheyne-Stokes respiration and obstructive sleep apnoea are independent risk factors for malignant ventricular arrhythmias requiring appropriate cardioverter-defibrillator therapies in patients with congestive heart failure. Eur Heart J 2011;32:61–74. 20846992 10.1093/eurheartj/ehq327

[162] Hoffstein V , Mateika S. Cardiac arrhythmias, snoring, and sleep apnea. Chest 1994;106:466–471. 7774322 10.1378/chest.106.2.466

[163] Mansukhani MP , Calvin AD, Kolla BP, Brown RD, Lipford MC, Somers VK, Caples SM. The association between atrial fibrillation and stroke in patients with obstructive sleep apnea: a population-based case-control study. Sleep Med 2013;14:243–246. 23340087 10.1016/j.sleep.2012.08.021 PMC3582732

[164] Lavergne F , Morin L, Armitstead J, Benjafield A, Richards G, Woehrle H. Atrial fibrillation and sleep-disordered breathing. J Thorac Dis 2015;7:E575–E584. 26793367 10.3978/j.issn.2072-1439.2015.12.57 PMC4703675

[165] Qureshi WT , Nasir UB, Alqalyoobi S, O'Neal WT, Mawri S, Sabbagh S, Soliman EZ, Al-Mallah MH. Meta-analysis of continuous positive airway pressure as a therapy of atrial fibrillation in obstructive sleep apnea. Am J Cardiol 2015;116:1767–1773. 26482182 10.1016/j.amjcard.2015.08.046

[166] Full KM , Lutsey PL, Norby FL, Alonso A, Soliman EZ, Rooney MR, Chen LY. Association between excessive daytime sleepiness and measures of supraventricular arrhythmia burden: evidence from the Atherosclerosis Risk in Communities (ARIC) study. Sleep Breath 2020;24:1223–1227. 32215831 10.1007/s11325-020-02046-9 PMC7931629

[167] Platek AE , Szymanski FM, Filipiak KJ, Dudzik-Plocica A, Krzowski B, Karpinski G. Stratification of cardiovascular risk in patients with atrial fibrillation and obstructive sleep apnea-validity of the 2MACE score. Sleep Breath 2017;21:601–606. 28155102 10.1007/s11325-017-1469-6 PMC5585292

[168] Yaranov DM , Smyrlis A, Usatii N, Butler A, Petrini JR, Mendez J, Warshofsky MK. Effect of obstructive sleep apnea on frequency of stroke in patients with atrial fibrillation. Am J Cardiol 2015;115:461–465. 25529543 10.1016/j.amjcard.2014.11.027

[169] Dalgaard F , North R, Pieper K, Fonarow GC, Kowey PR, Gersh BJ, Mahaffey KW, Pokorney S, Steinberg BA, Naccarrelli G, Allen LA, Reiffel JA, Ezekowitz M, Singer DE, Chan PS, Peterson ED, Piccini JP. Risk of major cardiovascular and neurologic events with obstructive sleep apnea among patients with atrial fibrillation. Am Heart J 2020;223:65–71. 32179257 10.1016/j.ahj.2020.01.001 PMC7214210

[170] McEvoy RD , Antic NA, Heeley E, Luo Y, Ou Q, Zhang X, Mediano O, Chen R, Drager LF, Liu Z, Chen G, Du B, McArdle N, Mukherjee S, Tripathi M, Billot L, Li Q, Lorenzi-Filho G, Barbe F, Redline S, Wang J, Arima H, Neal B, White DP, Grunstein RR, Zhong N, Anderson CS; SAVE Investigators and Coordinators. CPAP for prevention of cardiovascular events in obstructive sleep apnea. N Engl J Med 2016;375:919–931. 27571048 10.1056/NEJMoa1606599

[171] Jelic S , Le Jemtel TH. Inflammation, oxidative stress, and the vascular endothelium in obstructive sleep apnea. Trends Cardiovasc Med 2008;18:253–260. 19232954 10.1016/j.tcm.2008.11.008

[172] Khalyfa A , Gozal D. Connexins and atrial fibrillation in obstructive sleep apnea. Curr Sleep Med Rep 2018;4:300–311. 31106116 10.1007/s40675-018-0130-7 PMC6516763

[173] Charlson ME , Pompei P, Ales KL, MacKenzie CR. A new method of classifying prognostic comorbidity in longitudinal studies: development and validation. J Chronic Dis 1987;40:373–383. 3558716 10.1016/0021-9681(87)90171-8

[174] Elixhauser A , Steiner C, Harris DR, Coffey RM. Comorbidity measures for use with administrative data. Med Care 1998;36:8–27. 9431328 10.1097/00005650-199801000-00004

[175] Proietti M , Marzona I, Vannini T, Tettamanti M, Fortino I, Merlino L, Basili S, Mannucci PM, Boriani G, Lip GYH, Roncaglioni MC, Nobili A. Long-term relationship between atrial fibrillation, multimorbidity and oral anticoagulant drug use. Mayo Clin Proc 2019;94:2427–2436. 31668449 10.1016/j.mayocp.2019.06.012

[176] Dagres N , Chao T-F, Fenelon G, Aguinaga L, Benhayon D, Benjamin EJ, Bunch TJ, Chen LY, Chen S-A, Darrieux F, de Paola A, Fauchier L, Goette A, Kalman J, Kalra L, Kim Y-H, Lane DA, Lip GYH, Lubitz SA, Márquez MF, Potpara T, Pozzer DL, Ruskin JN, Savelieva I, Teo WS, Tse H-F, Verma A, Zhang S, Chung MK, Bautista-Vargas W-F, Chiang C-E, Cuesta A, Dan G-A, Frankel DS, Guo Y, Hatala R, Lee YS, Murakawa Y, Pellegrini CN, Pinho C, Milan DJ, Morin DP, Nadalin E, Ntaios G, Prabhu MA, Proietti M, Rivard L, Valentino M, Shantsila A; ESC Scientific Document Group. European Heart Rhythm Association (EHRA)/Heart Rhythm Society (HRS)/Asia Pacific Heart Rhythm Society (APHRS)/Latin American Heart Rhythm Society (LAHRS) expert consensus on arrhythmias and cognitive function: what is the best practice? Europace 2018;20:1399–1421. 29562326 10.1093/europace/euy046 PMC6658813

[177] Malavasi VL , Zoccali C, Brandi MC, Micali G, Vitolo M, Imberti JF, Mussi C, Schnabel RB, Freedman B, Boriani G. Cognitive impairment in patients with atrial fibrillation: implications for outcome in a cohort study. Int J Cardiol 2020;323:83–89. 32800908 10.1016/j.ijcard.2020.08.028

[178] Proietti M , Cesari M. Frailty: what is it? Adv Exp Med Biol 2020;1216:1–7. 31894541 10.1007/978-3-030-33330-0_1

[179] Gugganig R , Aeschbacher S, Leong DP, Meyre P, Blum S, Coslovsky M, Beer JH, Moschovitis G, Müller D, Anker D, Rodondi N, Stempfel S, Mueller C, Meyer-Zürn C, Kühne M, Conen D, Osswald S; for the Swiss-AF Investigators. Frailty to predict unplanned hospitalization, stroke, bleeding, and death in atrial fibrillation. Eur Heart J Qual Care Clin Outcomes 2021;7:42–51. 31977016 10.1093/ehjqcco/qcaa002

[180] Villani ER , Tummolo AM, Palmer K, Gravina EM, Vetrano DL, Bernabei R, Onder G, Acampora N. Frailty and atrial fibrillation: a systematic review. Eur J Intern Med 2018;56:33–38. 29936074 10.1016/j.ejim.2018.04.018

[181] Wilkinson C , Todd O, Clegg A, Gale CP, Hall M. Management of atrial fibrillation for older people with frailty: a systematic review and meta-analysis. Age Ageing 2019;48:196–203. 30445608 10.1093/ageing/afy180 PMC6424377

[182] Madhavan M , Holmes DN, Piccini JP, Ansell JE, Fonarow GC, Hylek EM, Kowey PR, Mahaffey KW, Thomas L, Peterson ED, Chan P, Allen LA, Gersh BJ; ORBIT AF Investigators. Association of frailty and cognitive impairment with benefits of oral anticoagulation in patients with atrial fibrillation. Am Heart J 2019;211:77–89. 30901602 10.1016/j.ahj.2019.01.005

[183] Yang PS , Sung JH, Jang E, Yu HT, Kim TH, Lip GYH, Joung B. Application of the simple atrial fibrillation better care pathway for integrated care management in frail patients with atrial fibrillation: a nationwide cohort study. J Arrhythmia 2020;36:668–677. 10.1002/joa3.12364 PMC7411200 32782638

[184] Wilson JL , Altman RB. Biomarkers: delivering on the expectation of molecularly driven, quantitative health. Exp Biol Med (Maywood) 2018;243:313–322. 29199461 10.1177/1535370217744775 PMC5813871

[185] Infante T , Francone M, De Rimini ML, Cavaliere C, Canonico R, Catalano C, Napoli C. Machine learning and network medicine: a novel approach for precision medicine and personalized therapy in cardiomyopathies. J Cardiovasc Med (Hagerstown) 2020;22:429–440. 10.2459/JCM.0000000000001103 32890235

[186] Boriani G , Proietti M. Screening for atrial fibrillation: need for an integrated, structured approach. Eur J Intern Med 2019;67:33–35. 31375253 10.1016/j.ejim.2019.07.017

[187] Boriani G , Palmisano P, Malavasi VL, Fantecchi E, Vitolo M, Bonini N, Imberti JF, Valenti AC, Schnabel RB, Freedman B. Clinical factors associated with atrial fibrillation detection on single-time point screening using a hand-held single-lead ECG device. J Clin Med 2021;10:729. 33673209 10.3390/jcm10040729 PMC7917757

[188] Nattel S , Heijman J, Zhou L, Dobrev D. Molecular basis of atrial fibrillation pathophysiology and therapy: a translational perspective. Circ Res 2020;127:51–72. 32717172 10.1161/CIRCRESAHA.120.316363 PMC7398486

[189] Schotten U , Hatem S, Ravens U, Jaïs P, Müller FU, Goette A, Rohr S, Antoons G, Pieske B, Scherr D, Oto A, Casadei B, Verheule S, Cartlidge D, Steinmeyer K, Götsche T, Dobrev D, Kockskämper J, Lendeckel U, Fabritz L, Kirchhof P, Camm AJ; EUTRAF investigators. The European Network for Translational Research in Atrial Fibrillation (EUTRAF): objectives and initial results. Europace 2015;17:1457–1466. 26364316 10.1093/europace/euv252

[190] Guo Y , Guo J, Shi X, Yao Y, Sun Y, Xia Y, Yu B, Liu T, Chen Y, Lip GYH, Investigators M-A. Mobile health technology-supported atrial fibrillation screening and integrated care: a report from the mAFA-II trial Long-term Extension Cohort. Eur J Intern Med 2020;82:105–111. 33067121 10.1016/j.ejim.2020.09.024 PMC7553102

[191] Hermans ANL , van der Velden RMJ, Gawalko M, Verhaert DVM, Desteghe L, Duncker D, Manninger M, Heidbuchel H, Pisters R, Hemels M, Pison L, Sohaib A, Sultan A, Steven D, Wijtvliet P, Tieleman R, Gupta D, Dobrev D, Svennberg E, Crijns HJGM, Pluymaekers NAHA, Hendriks JM, Linz D; TeleCheck-AF investigators. On-demand mobile health infrastructures to allow comprehensive remote atrial fibrillation and risk factor management through teleconsultation. Clin Cardiol 2020;43:1232–1239. 33030259 10.1002/clc.23469 PMC7661648

[192] Pluymaekers NAHA , Hermans ANL, van der Velden RMJ, Gawałko M, den Uijl DW, Buskes S, Vernooy K, Crijns HJGM, Hendriks JM, Linz D. Implementation of an on-demand app-based heart rate and rhythm monitoring infrastructure for the management of atrial fibrillation through teleconsultation: teleCheck-AF. Europace 2020;23:345–352. 10.1093/europace/euaa201 PMC7499572 32887994

[193] Martin CM , Félix-Bortolotti M. W(h)ither complexity? The emperor's new toolkit? Or elucidating the evolution of health systems knowledge? J Eval Clin Pract 2010;16:415–420. 20604821 10.1111/j.1365-2753.2010.01461.x

[194] Inohara T , Shrader P, Pieper K, Blanco RG, Thomas L, Singer DE, Freeman JV, Allen LA, Fonarow GC, Gersh B, Ezekowitz MD, Kowey PR, Reiffel JA, Naccarelli GV, Chan PS, Steinberg BA, Peterson ED, Piccini JP. Association of atrial fibrillation clinical phenotypes with treatment patterns and outcomes: a multicenter registry study. JAMA Cardiol 2018;3:54–63. 29128866 10.1001/jamacardio.2017.4665 PMC5833527

