# Publisher’s note to: Optimizing indices of atrial fibrillation susceptibility and burden to evaluate atrial fibrillation severity, risk and outcomes

**DOI:** 10.1093/cvr/cvab361

**Published:** 2022-01-05

**Authors:** Giuseppe Boriani, Marco Vitolo, Igor Diemberger, Marco Proietti, Anna Chiara Valenti, Vincenzo Livio Malavasi, Gregory Y H Lip

*Cardiovascular Research*, cvab147, https://doi.org/10.1093/cvr/cvab147

This article should have published into Issue 117-7 of *Cardiovascular Research* as online-only content. However, it was accidentally omitted. The article has been added to the issue retrospectively and we have published this notice to highlight the emendation.

