# Supplementary material for: Optimizing indices of atrial fibrillation susceptibility and burden to evaluate atrial fibrillation severity, risk and outcomes
Source: Cardiovasc Res. 2021 Apr 29;117(7):1–21. doi: 10.1093/cvr/cvab147 (PMC8707734; doi:10.1093/cvr/cvab147)
Supplement: cvab147_Supplementary_Material [file cvab147_supplementary_material.docx]

**SUPPLEMENTARY MATERIAL**

**Optimizing indices of AF susceptibility and burden to**

**evaluate AF severity, risk and outcomes**

Giuseppe Boriani^1*^, Marco Vitolo^1,2,3^, Igor Diemberger^4^, Marco Proietti^2,5,6^, Anna Chiara Valenti^1^, Vincenzo Livio Malavasi^1^, Gregory YH Lip^2,7^

(1) Cardiology Division, Department of Biomedical, Metabolic and Neural Sciences, University of Modena and Reggio Emilia, Policlinico di Modena, Modena, Italy.

(2) Liverpool Centre for Cardiovascular Science, University of Liverpool and Liverpool Heart & Chest Hospital, Liverpool, United Kingdom

(3) Clinical and Experimental Medicine PhD Program, University of Modena and Reggio Emilia, Modena, Italy

(4) Department of Experimental, Diagnostic and Specialty Medicine, Institute of Cardiology, University of Bologna, Policlinico S. Orsola-Malpighi, Bologna, Italy

(5) Department of Clinical Sciences and Community Health, University of Milan, Milan, Italy

(6) Geriatric Unit, IRCCS Istituti Clinico Scientifici Maugeri, Milan, Italy;

(7) Aalborg Thrombosis Research Unit, Department of Clinical Medicine, Aalborg University, Aalborg, Denmark.

**Words**: 9835

**Table**: 4

**Figure**: 3

**Short title**: AF susceptibility and burden

**^*^Corresponding Author:**

Prof. Giuseppe Boriani, MD, PhD

Cardiology Division, Department of Biomedical, Metabolic and Neural Sciences,

University of Modena and Reggio Emilia, Policlinico di Modena

Via del Pozzo, 71, 41124 Modena, Italy

*E-mail: giuseppe.boriani@unimore.it*

**Funding:** No funding was received for this work.

**TABLES’ REFERENCES**

**Table 2. Determinants of incident AF across large epidemiological population-based studies and impact on risk factors/comorbidities.**

**References (S1-S18)**

S1. Bild DE, Bluemke DA, Burke GL, Detrano R, Diez Roux AV, Folsom AR, Greenland P, Jacob DR, Kronmal R, Liu K, Nelson JC, O'Leary D, Saad MF, Shea S, Szklo M, Tracy RP. Multi-Ethnic Study of Atherosclerosis: objectives and design. *Am J Epidemiol* 2002;**156**:871-881.

S2. Habibi M, Samiei S, Ambale Venkatesh B, Opdahl A, Helle-Valle TM, Zareian M, Almeida AL, Choi EY, Wu C, Alonso A, Heckbert SR, Bluemke DA, Lima JA. Cardiac Magnetic Resonance-Measured Left Atrial Volume and Function and Incident Atrial Fibrillation: Results From MESA (Multi-Ethnic Study of Atherosclerosis). *Circulation Cardiovascular imaging* 2016;**9**.

S3. Patton KK, Heckbert SR, Alonso A, Bahrami H, Lima JA, Burke G, Kronmal RA. N-terminal pro-B-type natriuretic peptide as a predictor of incident atrial fibrillation in the Multi-Ethnic Study of Atherosclerosis: the effects of age, sex and ethnicity. *Heart (British Cardiac Society)* 2013;**99**:1832-1836.

S4. Lin GM, Colangelo LA, Lloyd-Jones DM, Redline S, Yeboah J, Heckbert SR, Nazarian S, Alonso A, Bluemke DA, Punjabi NM, Szklo M, Liu K. Association of Sleep Apnea and Snoring With Incident Atrial Fibrillation in the Multi-Ethnic Study of Atherosclerosis. *American journal of epidemiology* 2015;**182**:49-57.

S5. Chrispin J, Jain A, Soliman EZ, Guallar E, Alonso A, Heckbert SR, Bluemke DA, Lima JA, Nazarian S. Association of electrocardiographic and imaging surrogates of left ventricular hypertrophy with incident atrial fibrillation: MESA (Multi-Ethnic Study of Atherosclerosis). *J Am Coll Cardiol* 2014;**63**:2007-2013.

S6. O'Neal WT, Efird JT, Dawood FZ, Yeboah J, Alonso A, Heckbert SR, Soliman EZ. Coronary artery calcium and risk of atrial fibrillation (from the multi-ethnic study of atherosclerosis). *Am J Cardiol* 2014;**114**:1707-1712.

S7. O'Neal WT, Soliman EZ, Qureshi W, Alonso A, Heckbert SR, Herrington D. Sustained pre-hypertensive blood pressure and incident atrial fibrillation: the Multi-Ethnic Study of Atherosclerosis. *Journal of the American Society of Hypertension : JASH* 2015;**9**:191-196.

S8. Alonso A, Roetker NS, Soliman EZ, Chen LY, Greenland P, Heckbert SR. Prediction of Atrial Fibrillation in a Racially Diverse Cohort: The Multi-Ethnic Study of Atherosclerosis (MESA). *Journal of the American Heart Association* 2016;**5**.

S9. Heeringa J, van der Kuip DA, Hofman A, Kors JA, van Herpen G, Stricker BH, Stijnen T, Lip GY, Witteman JC. Prevalence, incidence and lifetime risk of atrial fibrillation: the Rotterdam study. *Eur Heart J* 2006;**27**:949-953.

S10. Ott A, Breteler MM, de Bruyne MC, van Harskamp F, Grobbee DE, Hofman A. Atrial fibrillation and dementia in a population-based study. The Rotterdam Study. *Stroke; a journal of cerebral circulation* 1997;**28**:316-321.

S11. Benjamin EJ, Levy D, Vaziri SM, D'Agostino RB, Belanger AJ, Wolf PA. Independent risk factors for atrial fibrillation in a population-based cohort. The Framingham Heart Study. *JAMA : the journal of the American Medical Association* 1994;**271**:840-844.

S12. Lloyd-Jones DM, Wang TJ, Leip EP, Larson MG, Levy D, Vasan RS, D'Agostino RB, Massaro JM, Beiser A, Wolf PA, Benjamin EJ. Lifetime risk for development of atrial fibrillation: the Framingham Heart Study. *Circulation* 2004;**110**:1042-1046.

S13. Alonso A, Lopez FL, Matsushita K, Loehr LR, Agarwal SK, Chen LY, Soliman EZ, Astor BC, Coresh J. Chronic kidney disease is associated with the incidence of atrial fibrillation: the Atherosclerosis Risk in Communities (ARIC) study. *Circulation* 2011;**123**:2946-2953.

S14. Chamberlain AM, Agarwal SK, Folsom AR, Soliman EZ, Chambless LE, Crow R, Ambrose M, Alonso A. A clinical risk score for atrial fibrillation in a biracial prospective cohort (from the Atherosclerosis Risk in Communities [ARIC] study). *The American journal of cardiology* 2011;**107**:85-91.

S15. Rosenberg MA, Patton KK, Sotoodehnia N, Karas MG, Kizer JR, Zimetbaum PJ, Chang JD, Siscovick D, Gottdiener JS, Kronmal RA, Heckbert SR, Mukamal KJ. The impact of height on the risk of atrial fibrillation: the Cardiovascular Health Study. *European heart journal* 2012;**33**:2709-2717.

S16. Jensen PN, Thacker EL, Dublin S, Psaty BM, Heckbert SR. Racial differences in the incidence of and risk factors for atrial fibrillation in older adults: the cardiovascular health study. *Journal of the American Geriatrics Society* 2013;**61**:276-280.

S17. Psaty BM, Manolio TA, Kuller LH, Kronmal RA, Cushman M, Fried LP, White R, Furberg CD, Rautaharju PM. Incidence of and risk factors for atrial fibrillation in older adults. *Circulation* 1997;**96**:2455-2461.

S18. Krahn AD, Manfreda J, Tate RB, Mathewson FA, Cuddy TE. The natural history of atrial fibrillation: incidence, risk factors, and prognosis in the Manitoba Follow-Up Study. *The American journal of medicine* 1995;**98**:476-484.

**Table 3. Impact of comorbidities on AF-associated outcomes, according to real-world registries on AF patients**

**References (S19-S42)**

S19. Piccini JP, Fraulo ES, Ansell JE, Fonarow GC, Gersh BJ, Go AS, Hylek EM, Kowey PR, Mahaffey KW, Thomas LE, Kong MH, Lopes RD, Mills RM, Peterson ED. Outcomes registry for better informed treatment of atrial fibrillation: rationale and design of ORBIT-AF. *American heart journal* 2011;**162**:606-612.e601.

S20. Vemulapalli S, Inohara T, Kim S, Thomas L, Piccini JP, Patel MR, Chang P, Fonarow GC, Ezekowitz MD, Hylek E, Go AS, Kowey PR, Mahaffey KW, Gersh BJ, Peterson ED. Blood Pressure Control and Cardiovascular Outcomes in Patients With Atrial Fibrillation (From the ORBIT-AF Registry). *Am J Cardiol* 2019;**123**:1628-1636.

S21. Steinberg BA, Blanco RG, Ollis D, Kim S, Holmes DN, Kowey PR, Fonarow GC, Ansell J, Gersh B, Go AS, Hylek E, Mahaffey KW, Thomas L, Chang P, Peterson ED, Piccini JP, Investigators O-ASC. Outcomes registry for better informed treatment of atrial fibrillation II: Rationale and design of the ORBIT-AF II registry. *American heart journal* 2014;**168**:160-167.

S22. Echouffo-Tcheugui JB, Shrader P, Thomas L, Gersh BJ, Kowey PR, Mahaffey KW, Singer DE, Hylek EM, Go AS, Peterson ED, Piccini JP, Fonarow GC. Care Patterns and Outcomes in Atrial Fibrillation Patients With and Without Diabetes: ORBIT-AF Registry. *Journal of the American College of Cardiology* 2017;**70**:1325-1335.

S23. Pandey A, Gersh BJ, McGuire DK, Shrader P, Thomas L, Kowey PR, Mahaffey KW, Hylek E, Sun S, Burton P, Piccini J, Peterson E, Fonarow GC. Association of Body Mass Index With Care and Outcomes in Patients With Atrial Fibrillation: Results From the ORBIT-AF Registry. *JACC Clin Electrophysiol* 2016;**2**:355-363.

S24. Steinberg BA, Kim S, Fonarow GC, Thomas L, Ansell J, Kowey PR, Mahaffey KW, Gersh BJ, Hylek E, Naccarelli G, Go AS, Reiffel J, Chang P, Peterson ED, Piccini JP. Drivers of hospitalization for patients with atrial fibrillation: Results from the Outcomes Registry for Better Informed Treatment of Atrial Fibrillation (ORBIT-AF). *American heart journal* 2014;**167**:735-742.e732.

S25. Cherian TS, Shrader P, Fonarow GC, Allen LA, Piccini JP, Peterson ED, Thomas L, Kowey PR, Gersh BJ, Mahaffey KW. Effect of Atrial Fibrillation on Mortality, Stroke Risk, and Quality-of-Life Scores in Patients With Heart Failure (from the Outcomes Registry for Better Informed Treatment of Atrial Fibrillation [ORBIT-AF]). *The American journal of cardiology* 2017;**119**:1763-1769.

S26. Inohara T, Shrader P, Pieper K, Blanco RG, Allen LA, Fonarow GC, Gersh BJ, Go AS, Ezekowitz MD, Kowey PR, Reiffel JA, Naccarelli GV, Chan PS, Mahaffey KW, Singer DE, Freeman JV, Steinberg BA, Peterson ED, Piccini JP, Investigators OAPa. Treatment of atrial fibrillation with concomitant coronary or peripheral artery disease: Results from the outcomes registry for better informed treatment of atrial fibrillation II. *Am Heart J* 2019;**213**:81-90.

S27. Kakkar AK, Mueller I, Bassand JP, Fitzmaurice DA, Goldhaber SZ, Goto S, Haas S, Hacke W, Lip GY, Mantovani LG, Verheugt FW, Jamal W, Misselwitz F, Rushton-Smith S, Turpie AG. International longitudinal registry of patients with atrial fibrillation at risk of stroke: Global Anticoagulant Registry in the FIELD (GARFIELD). *American heart journal* 2012;**163**:13-19.e11.

S28. Bassand JP, Accetta G, Camm AJ, Cools F, Fitzmaurice DA, Fox KA, Goldhaber SZ, Goto S, Haas S, Hacke W, Kayani G, Mantovani LG, Misselwitz F, Ten Cate H, Turpie AG, Verheugt FW, Kakkar AK, Investigators G-A. Two-year outcomes of patients with newly diagnosed atrial fibrillation: results from GARFIELD-AF. *Eur Heart J* 2016;**37**:2882-2889.

S29. Bassand JP, Accetta G, Al Mahmeed W, Corbalan R, Eikelboom J, Fitzmaurice DA, Fox KAA, Gao H, Goldhaber SZ, Goto S, Haas S, Kayani G, Pieper K, Turpie AGG, van Eickels M, Verheugt FWA, Kakkar AK, Investigators G-A. Risk factors for death, stroke, and bleeding in 28,628 patients from the GARFIELD-AF registry: Rationale for comprehensive management of atrial fibrillation. *PloS one* 2018;**13**:e0191592.

S30. Violi F, Daví G, Hiatt W, Lip GY, Corazza GR, Perticone F, Proietti M, Pignatelli P, Vestri AR, Basili S, Investigators AS. Prevalence of peripheral artery disease by abnormal ankle-brachial index in atrial fibrillation: implications for risk and therapy. *J Am Coll Cardiol* 2013;**62**:2255-2256.

S31. Raparelli V, Pastori D, Pignataro SF, Vestri AR, Pignatelli P, Cangemi R, Proietti M, Davì G, Hiatt WR, Lip GYH, Corazza GR, Perticone F, Violi F, Basili S, Collaborators AS. Major adverse cardiovascular events in non-valvular atrial fibrillation with chronic obstructive pulmonary disease: the ARAPACIS study. *Intern Emerg Med* 2018;**13**:651-660.

S32. Violi F, Davì G, Proietti M, Pastori D, Hiatt WR, Corazza GR, Perticone F, Pignatelli P, Farcomeni A, Vestri AR, Lip GY, Basili S, Investigators AAFRfA-BIPA-CISS. Ankle-Brachial Index and cardiovascular events in atrial fibrillation. The ARAPACIS Study. *Thromb Haemost* 2016;**115**:856-863.

S33. Pastori D, Pignatelli P, Perticone F, Sciacqua A, Carnevale R, Farcomeni A, Basili S, Corazza GR, Davì G, Lip GYH, Violi F, group AAFRfA-BIPA-CISs. Aspirin and renal insufficiency progression in patients with atrial fibrillation and chronic kidney disease. *Int J Cardiol* 2016;**223**:619-624.

S34. Boriani G, Proietti M, Laroche C, Diemberger I, Popescu MI, Riahi S, Shantsila A, Dan GA, Tavazzi L, Maggioni AP, Lip GYH. Changes to oral anticoagulant therapy and risk of death over a 3-year follow-up of a contemporary cohort of European patients with atrial fibrillation final report of the EURObservational Research Programme on Atrial Fibrillation (EORP-AF) pilot general registry. *Int J Cardiol* 2018;**271**:68-74.

S35. Proietti M, Laroche C, Opolski G, Maggioni AP, Boriani G, Lip GYH, Investigators AGP. 'Real-world' atrial fibrillation management in Europe: observations from the 2-year follow-up of the EURObservational Research Programme-Atrial Fibrillation General Registry Pilot Phase. *Europace* 2017;**19**:722-733.

S36. Lip GY, Laroche C, Ioachim PM, Rasmussen LH, Vitali-Serdoz L, Petrescu L, Darabantiu D, Crijns HJ, Kirchhof P, Vardas P, Tavazzi L, Maggioni AP, Boriani G. Prognosis and treatment of atrial fibrillation patients by European cardiologists: one year follow-up of the EURObservational Research Programme-Atrial Fibrillation General Registry Pilot Phase (EORP-AF Pilot registry). *Eur Heart J* 2014;**35**:3365-3376.

S37. Boriani G, Laroche C, Diemberger I, Popescu MI, Rasmussen LH, Petrescu L, Crijns HJGM, Tavazzi L, Maggioni AP, Lip GYH. Glomerular filtration rate in patients with atrial fibrillation and 1-year outcomes. *Sci Rep* 2016;**6**:30271.

S38. Boriani G, Laroche C, Diemberger I, Fantecchi E, Meeder J, Kurpesa M, Baluta MM, Proietti M, Tavazzi L, Maggioni AP, Lip GYH, Investigators E-AGPR. Overweight and obesity in patients with atrial fibrillation: Sex differences in 1-year outcomes in the EORP-AF General Pilot Registry. *J Cardiovasc Electrophysiol* 2018;**29**:566-572.

S39. Fumagalli S, Said SA, Laroche C, Gabbai D, Boni S, Marchionni N, Boriani G, Maggioni AP, Musialik-Lydka A, Sokal A, Petersen J, Crijns HJGM, Lip GYH, Investigators E-AGPR. Management and prognosis of atrial fibrillation in diabetic patients: an EORP-AF General Pilot Registry report. *Eur Heart J Cardiovasc Pharmacother* 2018;**4**:172-179.

S40. Lip GY, Laroche C, Popescu MI, Rasmussen LH, Vitali-Serdoz L, Dan GA, Kalarus Z, Crijns HJ, Oliveira MM, Tavazzi L, Maggioni AP, Boriani G. Heart failure in patients with atrial fibrillation in Europe: a report from the EURObservational Research Programme Pilot survey on Atrial Fibrillation. *Eur J Heart Fail* 2015;**17**:570-582.

S41. Boriani G, Proietti M, Laroche C, Fauchier L, Marin F, Nabauer M, Potpara T, Dan GA, Kalarus Z, Diemberger I, Tavazzi L, Maggioni AP, Lip GYH, Investigators E-AL-TGR, Coordinators) SCN. Contemporary stroke prevention strategies in 11 096 European patients with atrial fibrillation: a report from the EURObservational Research Programme on Atrial Fibrillation (EORP-AF) Long-Term General Registry. *Europace* 2018;**20**:747-757.

S42. Boriani G, Proietti M, Laroche C, Fauchier L, Marin F, Nabauer M, Potpara T, Dan GA, Kalarus Z, Tavazzi L, Maggioni AP, Lip GYH, Investigators E-AL-TGR. Association between antithrombotic treatment and outcomes at 1-year follow-up in patients with atrial fibrillation: the EORP-AF General Long-Term Registry. *Europace* 2019;**21**:1013-1022.
